# Postoperative outcomes in CNS WHO grade 2 and 3 meningioma: a systematic review and meta-analysis

**DOI:** 10.1007/s00423-026-04081-8

**Published:** 2026-05-18

**Authors:** William H. Cook, Fareha Khalil, Danesh H. Sivanesan, Conor S. Gillespie, Elika Karvandi, Adel E. Helmy

**Affiliations:** 1 Division of Neurosurgery, Department of Clinical Neurosciences, University of Cambridge, Cambridge, UK; 2 School of Medicine, University of Glasgow, Glasgow, UK

**Keywords:** Meningioma, Survival, Recurrence, Systematic review, Meta-analysis

## Abstract

**Purpose:**

WHO grade 2 and 3 meningiomas account for approximately 20% of all meningiomas. The relatively lower incidence has resulted in limited representation of clinical outcomes in the literature, with only broad estimates of postoperative prognosis currently available. This study aimed to establish pooled incidence of recurrence and 5-year progression-free survival (PFS) and overall survival (OS) rates for grade 2 and 3 meningiomas.

**Methods:**

A PRISMA-compliant systematic review was prospectively registered (PROSPERO CRD42023441009). MEDLINE, EMBASE, and the Cochrane Library were searched from inception to September 2023. Eligible studies enrolled adults (≥ 16 years) with histologically confirmed WHO grade 2 or 3 cranial meningiomas who underwent surgery with or without adjuvant radiotherapy and/or stereotactic radiosurgery. The primary outcome was pooled annual incidence of recurrence; secondary outcomes were pooled 5-year PFS and OS, estimated using random-effects models.

**Results:**

Seventy-four studies met inclusion criteria (68 reporting on grade 2, 22 on grade 3 meningiomas), comprising 4,937 grade 2 and 253 grade 3 patients. The pooled annual incidence of recurrence was 6% (95% CI: 5–7%; I^2^ = 86%) for grade 2 and 10% (95% CI: 7–14%; I^2^ = 76%) for grade 3 meningiomas. For grade 2 meningiomas, pooled 5-year PFS and OS were 69% (95% CI: 63–75%; I^2^ = 79%) and 85% (95% CI: 78–89%; I^2^ = 72%), respectively. For grade 3 meningiomas, pooled 5-year PFS and OS were 18% (95% CI: 9–33%; I^2^ = 55%) and 39% (95% CI: 30–50%; I^2^ = 35%), respectively.

**Conclusion:**

This systematic review and meta-analysis provides pooled estimates of recurrence rate, 5-year PFS, and 5-year OS for WHO grade 2 and 3 meningiomas. Interpretation is constrained by the predominantly retrospective design of included studies and considerable clinical heterogeneity across the pooled cohorts.

**Supplementary Information:**

The online version contains supplementary material available at 10.1007/s00423-026-04081-8.

## Introduction

Meningiomas are the most common primary intracranial tumour with an incidence of 10.15 per 100,000 [1]. Meningiomas are histologically defined according to the World Health Organisation (WHO) as grade 1, 2 (atypical), or 3 (anaplastic) [2]. Among these subtypes, grades 2 and 3 constitute a relatively small fraction (20%) [3]. Consequently, the available literature for WHO grade 2 and 3 meningiomas is scarce.

Relative to their WHO grade 1 counterpart, grade 2 and 3 meningiomas exhibit aggressive biology, with a tendency to recur following treatment [4]. Indeed, this is reflected as an overall trend toward diminished progression-free (PFS) and overall survival (OS) [5]. However, exact estimates of recurrence, PFS, and OS rates are difficult to ascertain given the heterogeneity of the literature [3]. Published recurrence rates for grade 2 and 3 tumours range from 30 to 94% [4, 6–8]. Similarly, broad estimates of 5-year OS rates for grade 2 and 3 meningiomas are 69% and < 50%, respectively [9, 10].

An accurate understanding of recurrence probability is a critical prognostic tool in the management of WHO grade 2 and 3 meningiomas. Our primary objective was to survey the available literature and define estimates of tumour recurrence rates across WHO grade 2 and 3 intracranial meningiomas. Secondarily, an examination of PFS and OS was conducted.

## Methods

### Search strategy & selection criteria

We conducted a systematic review and meta-analysis according to the Preferred Reporting Items for Systematic Reviews and Meta-Analysis guidelines [11]. The review was registered in PROSPERO (CRD42023441009). In a change from the original protocol, we selected meningioma recurrence rate as primary outcome and 5-year progression-free survival and overall survival as secondary outcomes.

We searched MEDLINE, EMBASE, and the Cochrane database of systematic reviews for full-text articles published in English, between inception and September 2023 using keywords that were approved by a clinical librarian (Supplemental Digital Content, Appendix 1). Reference lists of included articles were checked for additional studies. Search terms used included “atypical adj6 meningioma*” and “malignant adj6 meningioma*”, where “adj6” denotes a proximity operator requiring terms to appear within six words of each other. The full Ovid MEDLINE, EMBASE, and Cochrane Library searches can be found in Supplemental Digital Content, Appendix 1.

We included studies of adults (≥ 16 years) with either primary central nervous system (CNS) WHO grade 2 or 3 meningiomas managed with surgery with or without adjuvant radiotherapy that reported recurrence rate with or without 5-year progression-free survival (PFS) and/or overall survival (OS). We excluded studies that were conference abstracts and studies published before January 2000 to exclude older versions of the CNS WHO classification system. We included studies of CNS WHO grade 2 and 3 meningiomas as defined by authors and classification systems available at the time of publication. Chemotherapy trials and trials of other systemic therapies were excluded. Study abstracts, animal studies, reviews, and case reports (less than five WHO grade 2 meningiomas or two grade 3 meningiomas reported) were excluded. Only papers published in English were included. Two independent reviewers (W.H.C. and F.K.) screened all titles and abstracts for eligibility. Disagreement was resolved with discussion and consensus, and when discussion failed to lead to consensus, a third researcher mediated (C.S.G.).

## Data extraction

Data extraction was completed in full and in duplicate by at least two authors per article. The following were gathered about included studies: (1) total population with CNS WHO grade 2 or 3 meningiomas; (2) number of patients with primary versus recurrent or transformed meningiomas; (3) median or mean follow-up time (years); (4) the total number of patients who developed recurrence during follow-up; (5) overall incidence of recurrence; (6) the incidence of recurrence per year of patient follow-up. We recorded whether studies reported recurrence definitions and if they did, what their definition of recurrence was, which was generally local radiological recurrence (Supplementary Tables 1–2). PFS was extracted as reported by individual studies; in most cases this represented time from surgery to radiological recurrence or progression, although some studies also included death as an endpoint. OS was defined as time from surgery to death of any cause. The median time to development of recurrence was extracted when available, as was median PFS and OS, and 1-, 3-, and 5-year PFS and OS rates.

## Quality assessment

Retrospective studies were classified according to the Newcastle Ottawa Scale [12]. Studies with total scores of ≤ 6 were classified to have a high risk of bias. For studies that were posthoc analyses of previous randomised controlled trial (RCT) data, we assessed the original trial publication from which data was extracted.

### Statistical analysis

For the meta-analysis, we used a random effects model with inverse variance weighting for pooled annual incidence of recurrence, and a generalised linear mixed model for meta-analysis of proportions for 5-year PFS and OS [13, 14]. WHO grade 2 and 3 meningioma were considered separately. Two or more included studies were required to perform the meta-analysis. We generated forest plots for incidence on the basis of a random effects model. For each random effects model, we tested heterogeneity using the maximum restricted likelihood estimator. Total heterogeneity and I^2^ characteristics were calculated. Publication bias was evaluated and presented as funnel plots.

Sensitivity analysis was performed to assess the effect of the following variables in our analysis: whether studies used a definition for meningioma recurrence and high risk of bias.

Data analysis of descriptive statistics, meta-analysis, and figure generation was performed using the packages ggplot2 and meta (metarate and metaprop functions) in RStudio (version 2024.09.1 + 394, R Foundation for Statistical Computing, Vienna, Austria).

## Results

### Systematic review and characteristics

**Fig. 1:**
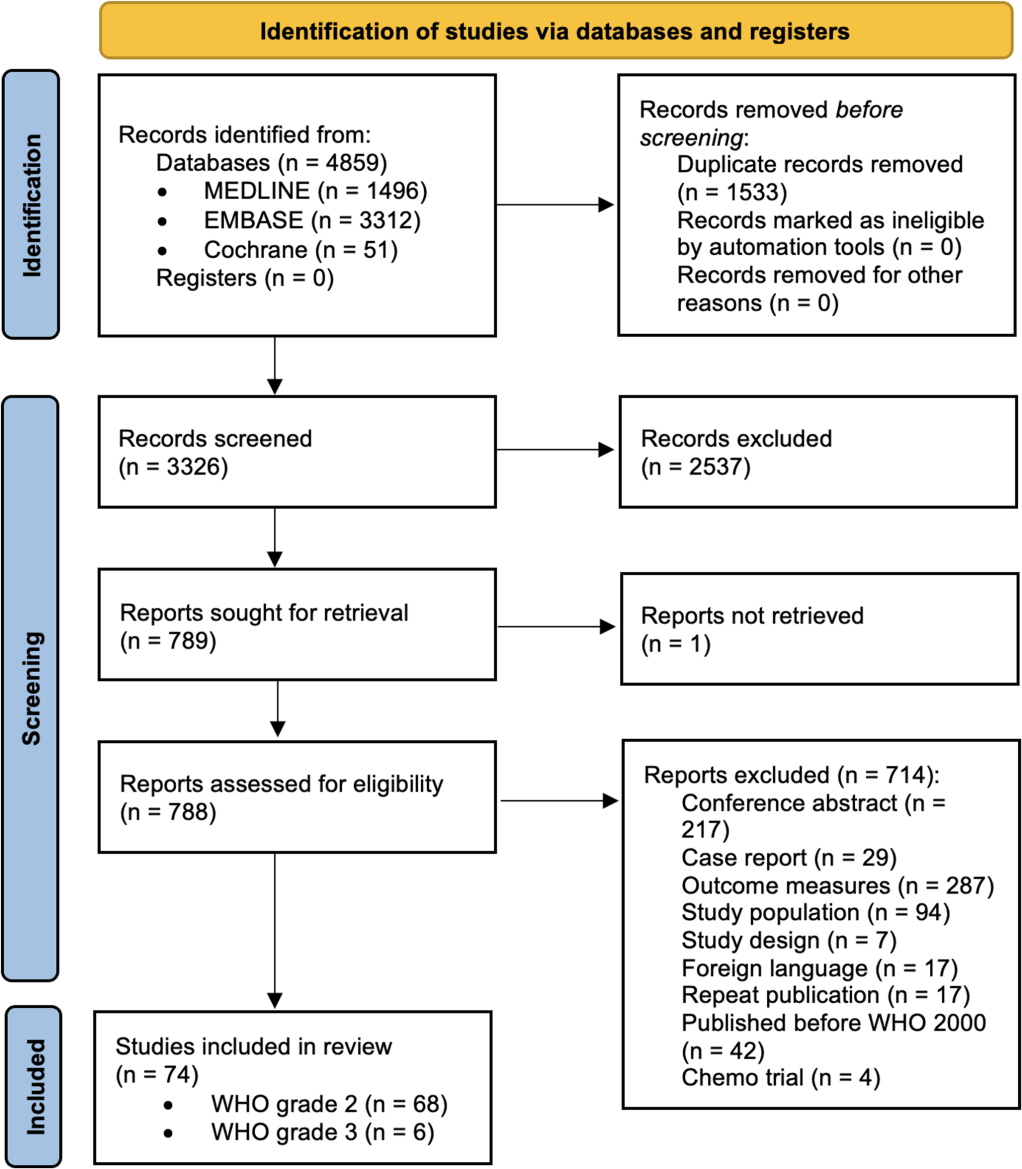
PRISMA 2020 flow diagram of database search, screening, and eligibility assessment for the present study

After full-text assessment, 74 studies (68 reporting on WHO grade 2 meningiomas, 22 on WHO grade 3 meningiomas) were assessed as suitable for inclusion. 714 studies were excluded at this stage (Fig. 1). Only one study of WHO grade 2 meningiomas was prospective [15], and the rest were retrospective (Supplemental Digital Content References). The same prospective study also reported on WHO grade 3 meningiomas [15]. All other studies of WHO grade 3 meningiomas were retrospective [16–36]. All included studies reported on primary meningiomas only or presented data for primary meningiomas separately to recurrent meningiomas. Five and three studies were published between 2000 and 2006 (corresponding to CNS WHO classification 3rd ed.) for grade 2 and 3 meningiomas respectively, 23 and eight between 2007 and 2015 (4th ed.), 22 and nine between 2016 and 2020 (4th revised), and 18 and two from 2021 onwards (5th ed.).

### Baseline characteristics

The baseline characteristics of included studies are presented in Supplemental Tables 1 and 2 (Supplemental Digital Content). The median number of patients included per study reporting on CNS WHO grade 2 meningiomas was 44.5 (4,937 total patients, interquartile range [IQR]: 22–100, range 6–523). Of these 68 included studies reporting recurrence rate, 15 studies (1698 patients) reported 5-year PFS and nine studies (864 patients) reported 5-year OS.

For CNS WHO grade 3 meningiomas, the median number of patients per study was 10.5 (253 total patients, IQR: 4.25–15.25, range 2–42). Of these 22 included studies reporting recurrence rate, four studies (81 patients) reported 5-year PFS and four studies (94 patients) reported 5-year OS.

The median follow-up period was 4.7 years (IQR: 3.7–5.9) and 5 years (IQR: 3.1–5.7) for studies of WHO grade 2 and 3 meningiomas, respectively.

### Pooled incidence of recurrence rate

**Fig. 2:**
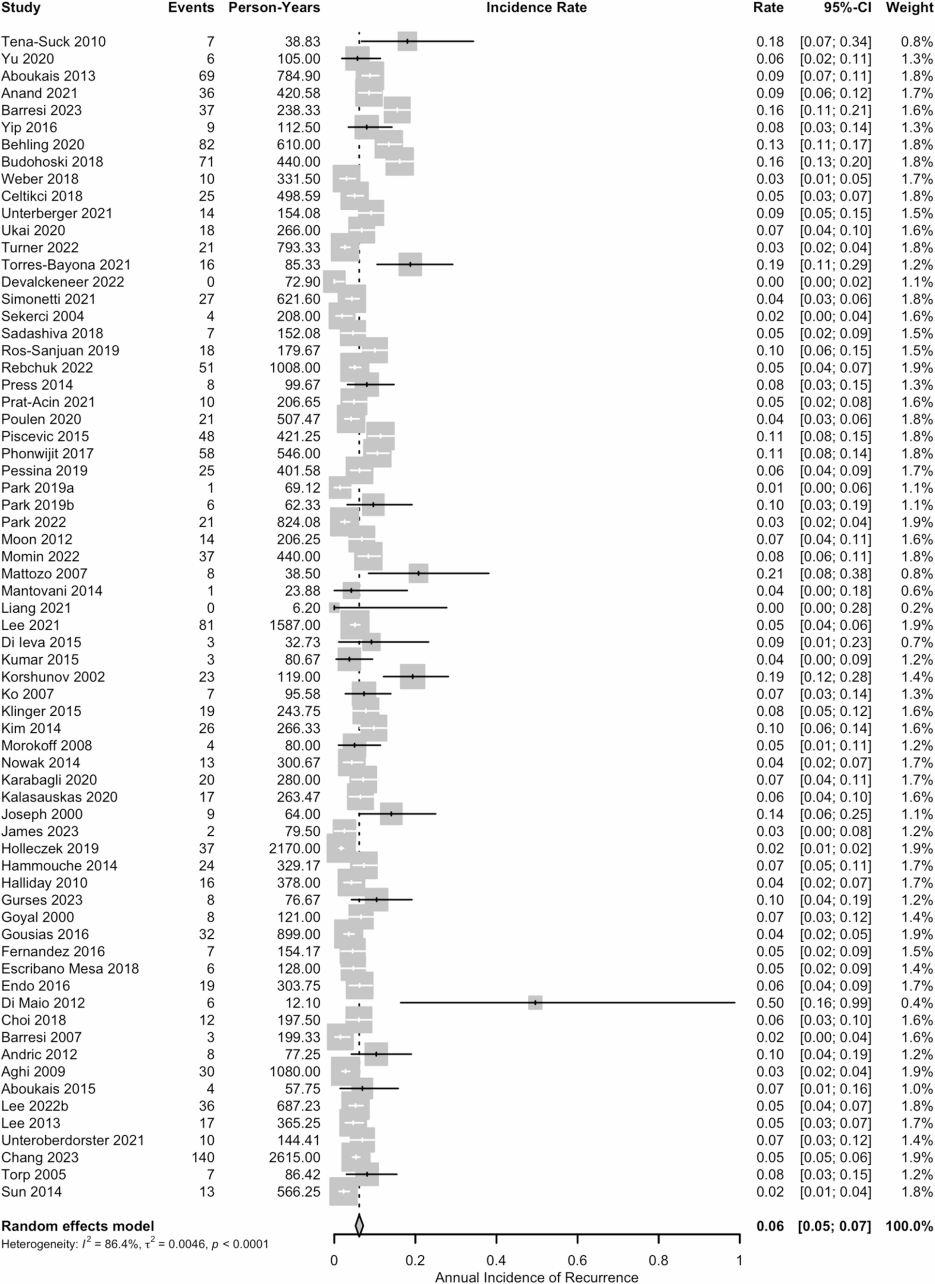
Forest plot of annual incidence of recurrence in WHO grade 2 meningioma. CI, confidence interval

**Fig. 3:**
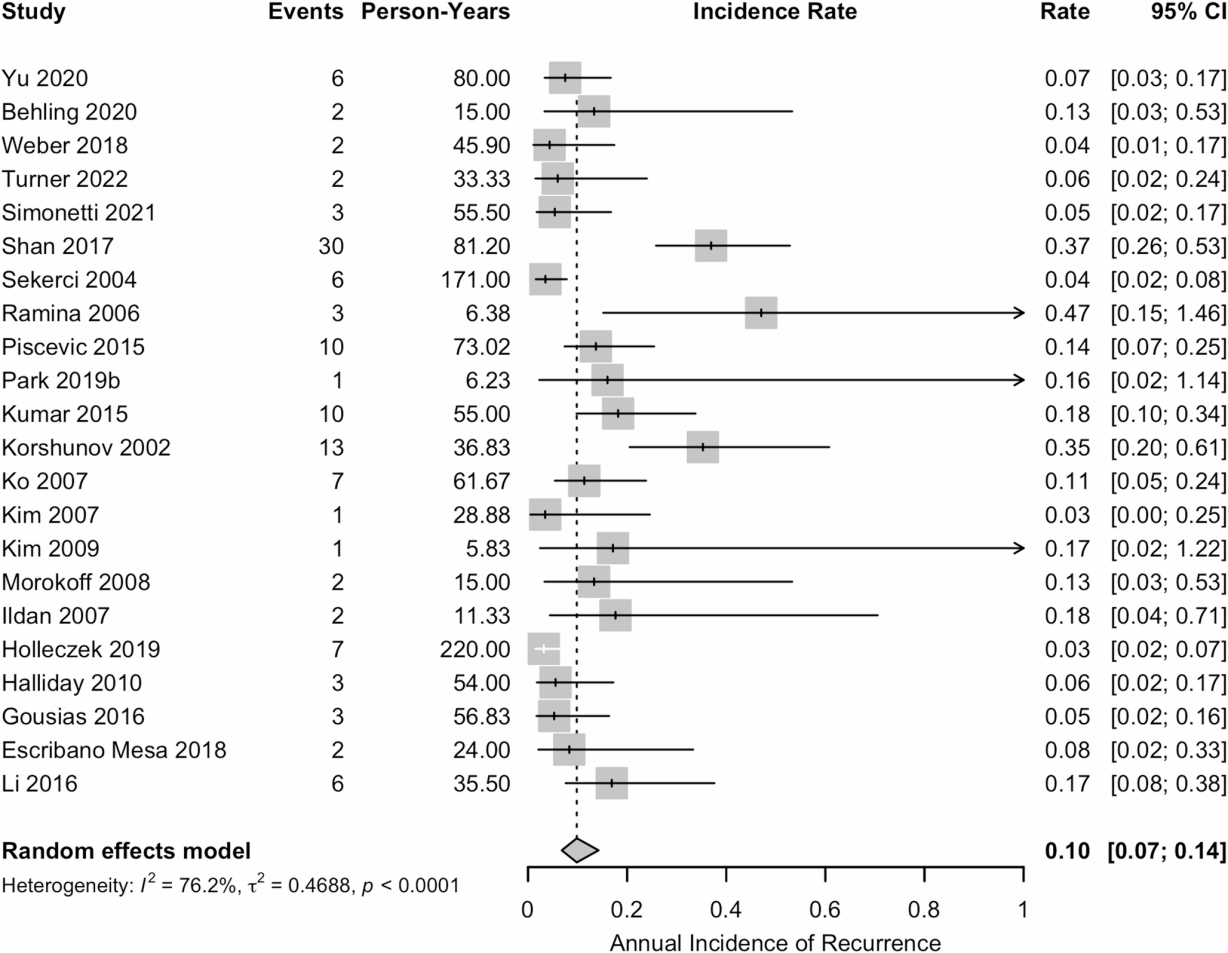
Forest plot of annual incidence of recurrence in WHO grade 3 meningioma. CI, confidence interval

The pooled annual incidence of recurrence of WHO grade 2 meningiomas was 6% (68 studies, 4,937 patients, 95% confidence interval [CI]: 5–7%, I^2^ = 86%) (Fig. 2). Pooled annual incidence of recurrence of WHO grade 3 meningiomas was 10% (22 studies, 253 patients, 95% CI: 7–14%, I^2^ = 76%) (Fig. 3).

### 5-year PFS

**Fig. 4:**
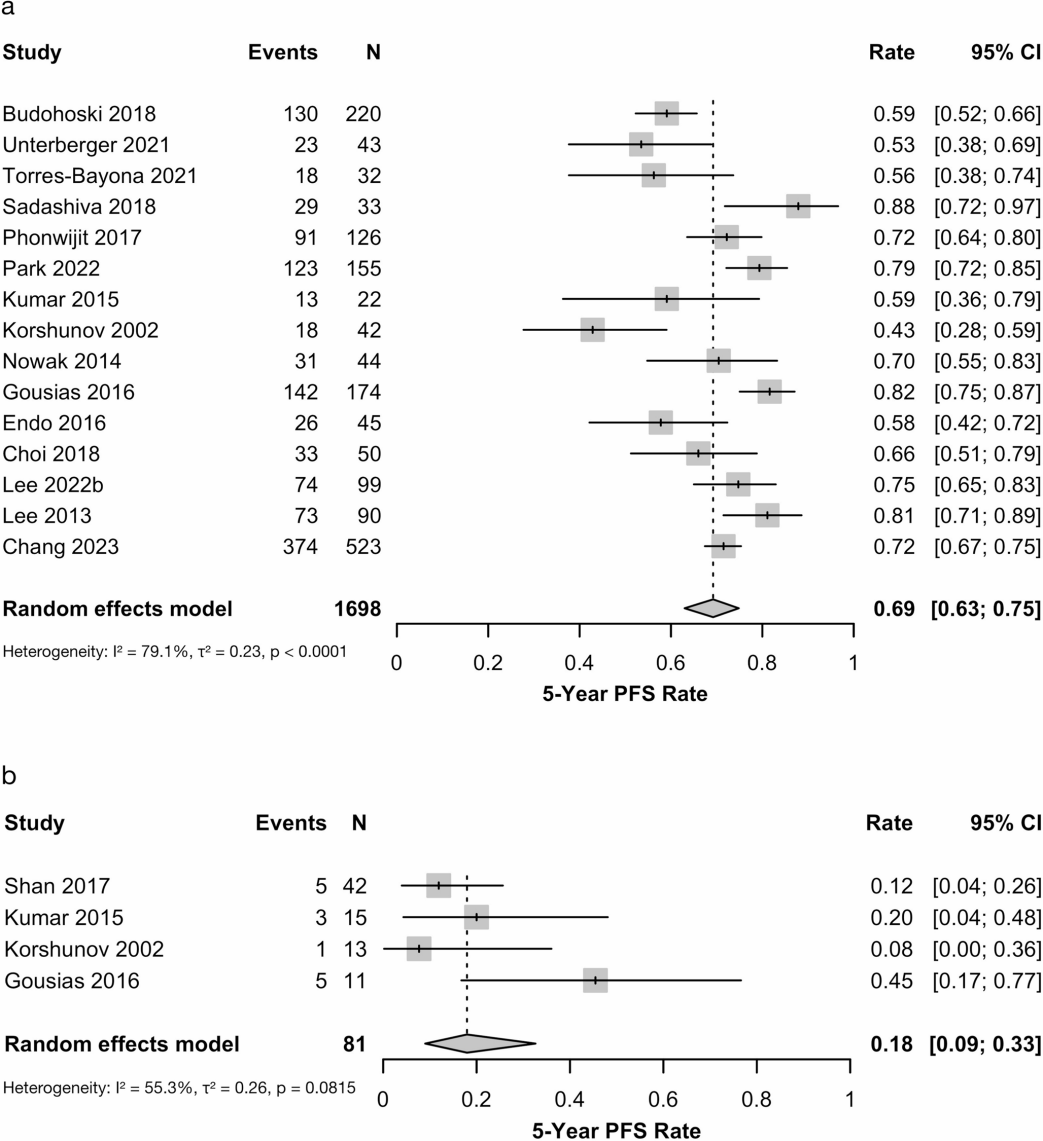
**A** Forest plot of 5-year progression-free survival (PFS) in WHO grade 2 meningioma. **B** Forest plot of 5-year PFS in WHO grade 3 meningioma. CI, confidence interval

Pooled 5-year PFS of WHO grade 2 meningiomas was 69% (15 studies, 1698 patients, 95% CI: 63–75%, I^2^ = 79%) (Fig. 4A) [29, 30, 35, 37–48]. Pooled 5-year PFS of WHO grade 3 meningiomas was 18% (4 studies, 81 patients, 95% CI: 9–33%, I^2^ = 55%) (Fig. 4B) [16, 29, 30, 35].

### 5-year OS

**Fig. 5:**
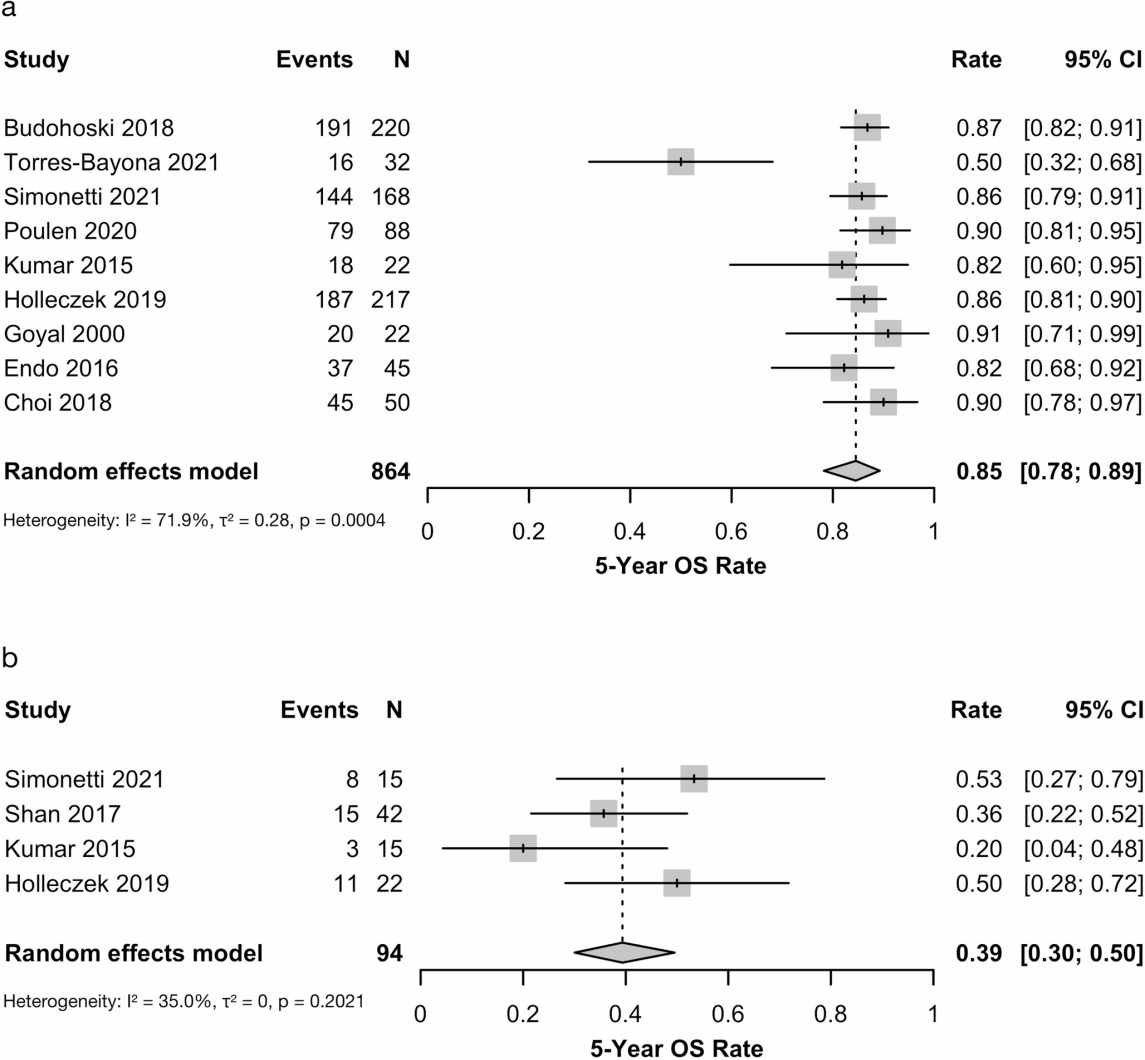
**A** Forest plot of 5-year overall survival (OS) in WHO grade 2 meningioma. **B** Forest plot of 5-year OS in WHO grade 3 meningioma. CI, confidence interval

Pooled 5-year OS of WHO grade 2 meningiomas was 85% (9 studies, 864 patients, 95% CI: 78–89%, I^2^ = 72%) (Fig. 5A) [25, 29, 33, 37, 39, 44, 45, 49, 50]. Pooled 5-year OS of WHO grade 3 meningiomas was 39% (4 studies, 94 patients, 95% CI: 30–50%, I^2^ = 35%) (Fig. 5B) [16, 25, 29, 33].

### Assessment of bias

The assessment of bias for retrospective cohort studies, using the Newcastle-Ottawa Scale, is detailed in Supplemental Fig. 1 (Supplemental Digital Content). The mean score for all studies was 7.4 (out of a maximum of 9), and 19 studies were classified as having a high risk of bias. The funnel plots for each forest plot generated are displayed in Supplemental Figs. 2–7 (Supplemental Digital Content). Asymmetry was present in most analyses, particularly in the WHO grade 2 plots where there were enough studies to be interpretable. The grade 3 plots were likely underpowered for reliable assessment due to low study numbers.

### Sensitivity analysis

The results of the 2-step sensitivity analysis are detailed in Supplemental Table 3 (Supplemental Digital Content). There were very small differences in the rates of annual incidence of recurrence in WHO grade 2 and 3 meningiomas when studies at high risk of bias or studies without recurrence definitions were removed. Removing studies without recurrence definitions decreased 5-year PFS of WHO grade 2 meningiomas to 65% (95% CI: 58–72%) from 69% (95% CI: 63–75%). Although study numbers were small, removing two studies of WHO grade 3 meningiomas without recurrence definitions decreased the 5-year OS of grade 3 meningioma to 32% (95% CI: 21–45%) from 39% (95% CI: 30–50%).

## Discussion

### Summary of findings

We performed a systematic review of three databases from inception to 2023 to pool studies of CNS WHO grade 2 and 3 meningiomas that had recurrence rate, PFS, and/or OS reported. We had 74 studies with data extracted to report on. To the best of our knowledge, this is the largest systematic review that evaluates the postoperative outcomes of CNS WHO grade 2 and 3 meningiomas. Of the 4,937 and 253 patients with WHO grade 2 and 3 meningiomas, respectively, pooled annual incidence of recurrence was 6% and 10% for grade 2 and 3 meningiomas, respectively. Five-year PFS was 69% and 18% and 5-year OS was 85% and 39% for grade 2 and 3 meningiomas, respectively.

While there are also no large systematic reviews of postoperative outcomes in WHO grade 1 meningiomas, the most recent Central Brain Tumor Registry of the United States report calculated that the 5-year OS of ‘non-malignant’ brain or other CNS tumours was 92% [1]. Most (56.8%) of these tumours were meningiomas [1]. It is well-established that WHO grade 2 and 3 meningiomas have a more aggressive clinical course than WHO grade 1 meningiomas. The emerging molecular classification and integrated risk assessment of meningioma has largely replaced traditional WHO grading in modern pathology departments and this information is being used to guide further observation and management [51, 52]. This classification system explains why some WHO grade 1 meningiomas recur and some grade 2 meningiomas do not [53]. Unfortunately this nomenclature is only beginning to enter the wider meningioma literature and there are few case series of patients who have this information available to pool.

### Tumour recurrence and survival

PFS rates quoted in the literature range widely from 16% [54] to 83% [55] and 8.7% to 61% [56] in WHO grade 2 and 3 meningiomas, respectively. This is likely due to the heterogeneity in patient selection, treatment course, follow-up schedule, and definition of recurrence. We have not attempted to stratify our results by proportion of patients with gross-total resection or proportion of patients receiving adjuvant radiotherapy. Adjuvant radiotherapy is indicated for grade 2 meningiomas following sub-total resection or grade 3 meningiomas [57]. An ongoing RCT, ‘ROAM’, is designed to determine whether adjuvant radiotherapy confers an improvement in radiological PFS for gross-totally resected grade 2 meningiomas [58]. In addition to reporting meningioma outcomes, there has been increasing interest in being able to predict specific outcomes based on tumour variables and treatment strategy, including with machine learning approaches [59].

A previous output from this systematic review demonstrated the poorly reported but likely worse health-related quality-of-life (HRQoL) of patients with WHO grade 2 or 3 meningiomas compared to grade 1 [60]. As with most meningioma literature, most studies and systematic reviews of HRQoL in meningioma have included mostly WHO grade 1 meningiomas [61]. Furthermore, while there is increasing evidence that meningioma patients suffer from long-term decreases in their HRQoL [62], it is unclear to what extent this is affected by the poorer prognosis of grade 2 and 3 meningiomas. There is some evidence that psychological domains of HRQoL are reduced in grade 2 and 3 meningioma compared to grade 1 [60]. Taken together, the present systematic review and meta-analysis of postoperative outcomes and similar studies of HRQoL demonstrate the need for more prospective studies of grade 2 and 3 meningiomas, stratified by treatments (extent of resection, adjuvant radiotherapy) and the use of a core outcome data set to collect data [63]. Nevertheless, the present meta-analysis provides clinicians and patients with estimated quantifications of annual incidence of recurrence, PFS, and OS for WHO grade 2 and 3 meningiomas.

### Limitations

This study has several limitations. All but one included study was retrospective and many studies were excluded at the full-text stage largely due to inappropriate outcome reporting or study population. Furthermore, we were interested in prognosis of primary grade 2 and 3 meningiomas, and many studies had to be excluded as they included a mixed population of primary and recurrent meningiomas and did not report outcomes separately. NF2 status was not a formal exclusion criterion. Patients with NF2 have an increased risk of grade 2 and 3 meningioma [57], and therefore NF2-associated disease may contribute to the pooled estimates to an unknown degree. There were also a number of non-English language studies excluded from the review. To obtain pooled estimates of annual incidence of recurrence, PFS, and OS, studies including patients with different extents of resection, rates and criteria for adjuvant radiotherapy, WHO classification eras, and surveillance practices were included. These are confounding variables that have a major impact on recurrence, PFS, and OS in WHO grade 2 and 3 meningiomas and could be assessed in future systematic reviews. Furthermore, included studies did not employ any molecular classification or integrated risk assessment systems for meningiomas which have been shown to be more predictive of meningioma outcome than WHO grading alone [51]. The choice of primary outcome, annual incidence of recurrence, is limited by a few factors. Firstly, recurrence in meningioma is heavily dependent on follow-up duration, imaging intervals, competing mortality, and treatment intensity. Converting this non-linear information into a pooled annual incidence may create an appearance of precision that is not fully supported by the data, especially as recurrence definitions vary substantially across studies. Nevertheless, we believe the outcome is a useful metric for patients and clinicians and has been reported in analogous studies.

The studies included in this systematic review demonstrated significant heterogeneity and most pooled measures demonstrated asymmetry in funnel plots, which likely reflects a combination of study heterogeneity and publication bias, which further reduces confidence in summary estimates. Furthermore, although average study quality was good, assessment of outcome was the greatest source of bias, which is a significant limitation for a systematic review of postoperative outcomes. However, we aimed to conduct a pragmatic systematic review that captured as many relevant studies as possible and performed random-effects meta-analysis to mitigate between-study heterogeneity.

## Conclusions

This systematic review and meta-analysis has evaluated key postoperative outcomes in CNS WHO grade 2 and 3 meningiomas. Figures for pooled annual incidence of recurrence, PFS, and OS will help clinicians and patients remain vigilant for long-term recurrence of meningiomas but are limited by nearly all studies being non-randomised retrospective studies and a small number of studies of grade 3 meningiomas. Extent of resection and adjuvant radiotherapy are likely the most important treatment-related determinants of recurrence and survival in grade 2 and 3 meningioma, and future work stratifying by these variables would be expected to demonstrate meaningfully different outcomes between treatment groups. Future work should also focus on improving the prospectively collected evidence base of grade 2 and 3 meningiomas and evaluating the prognosis of recurrent meningiomas separately.

## Supplementary Information

Below is the link to the electronic supplementary material.

Supplementary Material 1

Supplementary Material 2

Supplementary Material 3

Supplementary Material 4

## Data Availability

The datasets generated during and/or analysed during the current study are available from the corresponding author on reasonable request.

## References

[1] Price M, Ballard C, Benedetti J, Neff C, Cioffi G, Waite KA, Kruchko C, Barnholtz-Sloan JS, Ostrom QT (2024) CBTRUS statistical report: primary brain and other central nervous system tumors diagnosed in the United States in 2017–2021. Neuro Oncol 26:vi1–vi85. 10.1093/neuonc/noae145 39371035 10.1093/neuonc/noae145 PMC11456825

[2] Louis DN, Perry A, Wesseling P, Brat DJ, Cree IA, Figarella-Branger D, Hawkins C, Ng HK, Pfister SM, Reifenberger G, Soffietti R, Von Deimling A, Ellison DW (2021) The 2021 WHO classification of tumors of the central nervous system: a summary. Neuro Oncol 23:1231–1251. 10.1093/neuonc/noab106 34185076 10.1093/neuonc/noab106 PMC8328013

[3] Ostrom QT, Price M, Neff C, Cioffi G, Waite KA, Kruchko C, Barnholtz-Sloan JS (2022) CBTRUS statistical report: primary brain and other central nervous system tumors diagnosed in the United States in 2015–2019. Neuro Oncol 24:v1–v95. 10.1093/neuonc/noac202 36196752 10.1093/neuonc/noac202 PMC9533228

[4] Perry A, Scheithauer BW, Stafford SL, Lohse CM, Wollan PC (1999) “Malignancy” in meningiomas: a clinicopathologic study of 116 patients, with grading implications. Cancer 85:2046–2056. 10.1002/(SICI)1097-0142(19990501)85:9<2046::AID-CNCR23>3.0.CO;2-M 10.1002/(sici)1097-0142(19990501)85:9<2046::aid-cncr23>3.0.co;2-m 10223247

[5] Wang YC, Chuang CC, Wei KC, Chang CN, Lee ST, Wu CT, Hsu YH, Lin TK, Hsu PW, Huang YC, Tseng CK, Wang CC, Chen YL, Chen PY (2016) Long term surgical outcome and prognostic factors of atypical and malignant meningiomas. Sci Rep 6:35743. 10.1038/srep35743 27760993 10.1038/srep35743 PMC5071760

[6] Yang S, Park C, Park S, Kim D, Chung Y, Jung H (2008) Atypical and anaplastic meningiomas: prognostic implications of clinicopathological features. J Neurol Neurosurg Psychiatry 79:574–580. 10.1136/jnnp.2007.121582 17766430 10.1136/jnnp.2007.121582

[7] Pasquier D, Bijmolt S, Veninga T, Rezvoy N, Villa S, Krengli M, Weber DC, Baumert BG, Canyilmaz E, Yalman D, Szutowicz E, Tzuk-Shina T, Mirimanoff RO (2008) Atypical and malignant meningioma: outcome and prognostic factors in 119 irradiated patients. A multicenter, retrospective study of the Rare Cancer Network. Int J Radiat Oncol Biol Phys 71:1388–1393. 10.1016/j.ijrobp.2007.12.020 18294779 10.1016/j.ijrobp.2007.12.020

[8] Palma L, Celli P, Franco C, Cervoni L, Cantore G (1997) Long-term prognosis for atypical and malignant meningiomas: a study of 71 surgical cases. J Neurosurg 86:793–800. 10.3171/jns.1997.86.5.0793 9126894 10.3171/jns.1997.86.5.0793

[9] Recker MJ, Kuo CC, Prasad D, Attwood K, Plunkett RJ (2022) Incidence trends and survival analysis of atypical meningiomas: a population-based study from 2004 to 2018. J Neurooncol 160:13–22 35819682 10.1007/s11060-022-04085-6

[10] Combs SE, Schulz-Ertner D, Debus J, Von Deimling A, Hartmann C (2011) Improved correlation of the neuropathologic classification according to adapted World Health Organization classification and outcome after radiotherapy in patients with atypical and anaplastic meningiomas. Int J Radiat Oncol Biol Phys 81:1415–1421. 10.1016/j.ijrobp.2010.07.039 20932661 10.1016/j.ijrobp.2010.07.039

[11] Page MJ, McKenzie JE, Bossuyt PM, Boutron I, Hoffmann TC, Mulrow CD, Shamseer L, Tetzlaff JM, Akl EA, Brennan SE (2021) The PRISMA 2020 statement: an updated guideline for reporting systematic reviews. BMJ 372:n71 33782057 10.1136/bmj.n71 PMC8005924

[12] Stang A (2010) Critical evaluation of the Newcastle-Ottawa scale for the assessment of the quality of nonrandomized studies in meta-analyses. Eur J Epidemiol 25:603–605 20652370 10.1007/s10654-010-9491-z

[13] Schwarzer G, Rücker G (2022) Meta-analysis of proportions. Methods Mol Biol 2345:159–172 34550590 10.1007/978-1-0716-1566-9_10

[14] Lee KS, Zhang JJ, Nga VDW, Ng CH, Tai BC, Higgins JP, Syn NL (2022) Tenets for the proper conduct and use of meta-analyses: a practical guide for neurosurgeons. World Neurosurg 161:291-302.e291 35505547 10.1016/j.wneu.2021.09.034

[15] Weber DC, Ares C, Villa S, Peerdeman SM, Renard L, Baumert BG, Lucas A, Veninga T, Pica A, Jefferies S et al (2018) Adjuvant postoperative high-dose radiotherapy for atypical and malignant meningioma: a phase-II parallel non-randomized and observation study (EORTC 22042–26042). Radiother Oncol 128:260–265. 10.1016/j.radonc.2018.06.018 29960684 10.1016/j.radonc.2018.06.018

[16] Shan B, Zhang J, Song Y, Xu J (2017) Prognostic factors for patients with World Health Organization grade III meningiomas treated at a single center. Medicine (Baltimore) 96:e7385. 10.1097/MD.0000000000007385 28658170 10.1097/MD.0000000000007385 PMC5500092

[17] Ramina R, Neto MC, Fernandes YB, Aguiar PHP, De Meneses MS, Torres LFB (2006) Meningiomas of the jugular foramen. Neurosurg Rev 29:55–60. 10.1007/s10143-005-0415-4 16195869 10.1007/s10143-005-0415-4

[18] Kim EY, Weon YC, Kim ST, Kim HJ, Byun HS, Lee JI, Kim JH (2007) Rhabdoid meningioma: clinical features and MR imaging findings in 15 patients. AJNR Am J Neuroradiol 28:1462–1465. 10.3174/ajnr.A0601 17846191 10.3174/ajnr.A0601 PMC8134374

[19] Kim EY, Kim ST, Kim HJ, Jeon P, Kim KH, Byun HS (2009) Intraventricular meningiomas: radiological findings and clinical features in 12 patients. Clin Imaging 33:175–180. 10.1016/j.clinimag.2008.09.005 19411021 10.1016/j.clinimag.2008.09.005

[20] Ildan F, Erman T, Gocer AI, Tuna M, Bagdatoglu H, Cetinalp E, Burgut R (2007) Predicting the probability of meningioma recurrence in the preoperative and early postoperative period: a multivariate analysis in the midterm follow-up. Skull Base 17:157–171. 10.1055/s-2007-970554 17973029 10.1055/s-2007-970554 PMC1888737

[21] Li B, Tao B, Bai H, Zhong J, Wu X, Shi J, Sun H, Li S (2016) Papillary meningioma: an aggressive variant meningioma with clinical features and treatment: a retrospective study of 10 cases. Int J Neurosci 126:878–887. 10.3109/00207454.2015.1077833 26299848 10.3109/00207454.2015.1077833

[22] Yu J, Chen FF, Zhang HW, Zhang H, Luo SP, Huang GD, Lin F, Lei Y, Luo L (2020) Comparative analysis of the MRI characteristics of meningiomas according to the 2016 WHO pathological classification. Technol Cancer Res Treat. 10.1177/1533033820983287 10.1177/1533033820983287 PMC7768868 33356976

[23] Behling F, Fodi C, Gepfner-Tuma I, Machetanz K, Renovanz M, Skardelly M, Bornemann A, Honegger J, Tabatabai G, Tatagiba M, Schittenhelm J (2020) CNS invasion in meningioma—how the intraoperative assessment can improve the prognostic evaluation of tumor recurrence. Cancers (Basel) 12:3620. 10.3390/cancers12123620 33287241 10.3390/cancers12123620 PMC7761660

[24] Turner CP, McLay J, Hermans IF, Correia J, Bok A, Mehrabi N, Gock S, Highet B, Curtis MA, Dragunow M (2022) Tumour infiltrating lymphocyte density differs by meningioma type and is associated with prognosis in atypical meningioma. Pathology 54:417–424. 10.1016/j.pathol.2021.10.002 35082053 10.1016/j.pathol.2021.10.002

[25] Simonetti G, Silvani A, Tramacere I, Farinotti M, Legnani F, Pinzi V, Pollo B, Erbetta A, Gaviani P (2021) Long term follow up in 183 high grade meningioma: a single institutional experience. Clin Neurol Neurosurg 207:106808. 10.1016/j.clineuro.2021.106808 34293659 10.1016/j.clineuro.2021.106808

[26] Sekerci Z, Oral N, Ugurluoglu O, Colpan E, Ugur A (2004) Evaluation of forty-five atypical and malignant meningioma cases: over the 12-years follow-up period. Turk Neurosurg 14:12–20

[27] Piscevic I, Villa A, Milicevic M, Ilic R, Nikitovic M, Cavallo LM, Grujicic D (2015) The influence of adjuvant radiotherapy in atypical and anaplastic meningiomas: a series of 88 patients in a single institution. World Neurosurg 83:987–995. 10.1016/j.wneu.2015.02.021 25769488 10.1016/j.wneu.2015.02.021

[28] Park CK, Jung NY, Chang WS, Jung HH, Chang JW (2019) Gamma knife radiosurgery for postoperative remnant meningioma: analysis of recurrence factors according to World Health Organization grade. World Neurosurg 132:e399–e402. 10.1016/j.wneu.2019.08.136 31476462 10.1016/j.wneu.2019.08.136

[29] Kumar N, Kumar R, Khosla D, Salunke PS, Gupta SK, Radotra BD (2015) Survival and failure patterns in atypical and anaplastic meningiomas: a single-center experience of surgery and postoperative radiotherapy. J Cancer Res Ther 11:735–739. 10.4103/0973-1482.151426 26881510 10.4103/0973-1482.151426

[30] Korshunov A, Shishkina L, Golanov A (2002) DNA topoisomerase II-alpha and cyclin A immunoexpression in meningiomas and its prognostic significance: an analysis of 263 cases. Arch Pathol Lab Med 126:1079–1086 12204057 10.5858/2002-126-1079-DTIACA

[31] Ko KW, Nam DH, Kong DS, Lee JI, Park K, Kim JH (2007) Relationship between malignant subtypes of meningioma and clinical outcome. J Clin Neurosci 14:747–753. 10.1016/j.jocn.2006.05.005 17499508 10.1016/j.jocn.2006.05.005

[32] Morokoff AP, Zauberman J, Black PM (2008) Surgery for convexity meningiomas. Neurosurgery 63:427–434. 10.1227/01.NEU.0000310692.80289.28 18812953 10.1227/01.NEU.0000310692.80289.28

[33] Holleczek B, Zampella D, Urbschat S, Sahm F, von Deimling A, Oertel J, Ketter R (2019) Incidence, mortality and outcome of meningiomas: a population-based study from Germany. Cancer Epidemiol 62:101562. 10.1016/j.canep.2019.07.001 31325769 10.1016/j.canep.2019.07.001

[34] Halliday J, Fernandes H (2010) Meningioma recurrence: the efficacy and cost-effectiveness of current screening. Br J Neurosurg 24:55–61. 10.3109/02688690903431813 20158354 10.3109/02688690903431813

[35] Gousias K, Schramm J, Simon M (2016) The Simpson grading revisited: aggressive surgery and its place in modern meningioma management. J Neurosurg 125:551–560. 10.3171/2015.9.JNS15754 26824369 10.3171/2015.9.JNS15754

[36] Escribano Mesa JA, Alonso Morillejo E, Parron Carreno T, Huete Allut A, Narro Donate JM, Mendez Roman P, Contreras Jimenez A, Pedrero Garcia F, Masegosa Gonzalez J (2018) Risk of recurrence in operated parasagittal meningiomas: a logistic binary regression model. World Neurosurg 110:e112–e118. 10.1016/j.wneu.2017.10.087 29107168 10.1016/j.wneu.2017.10.087

[37] Budohoski KP, Clerkin J, Millward CP, O’Halloran PJ, Waqar M, Looby S, Young AMH, Guilfoyle MR, Fitzroll D, Devadass A, Allinson K, Farrell M, Javadpour M, Jenkinson MD, Santarius T, Kirollos RW (2018) Predictors of early progression of surgically treated atypical meningiomas. Acta Neurochir (Wien) 160:1813–1822. 10.1007/s00701-018-3593-x 29961125 10.1007/s00701-018-3593-x PMC6105233

[38] Unterberger A, Ng E, Pradhan A, Kondajji A, Kulinich D, Duong C, Yang I (2021) Adjuvant radiotherapy for atypical meningiomas is associated with improved progression free survival. J Neurol Sci 428:117590. 10.1016/j.jns.2021.117590 34358821 10.1016/j.jns.2021.117590

[39] Torres-Bayona S, Gil-Duran M, Rodriguez-Hernandez P, Monroy J, Africano P, Miranda-Acosta Y, Sampron N, Urculo E (2021) Radiotherapy versus observation after surgical resection of atypical meningiomas. Interdiscip Neurosurg 25:101201. 10.1016/j.inat.2021.101201

[40] Sadashiva N, Poyuran R, Mahadevan A, Bhat DI, Somanna S, Devi BI (2018) Chordoid meningioma: a clinico-pathological study of an uncommon variant of meningioma. J Neurooncol 137:575–582. 10.1007/s11060-018-2748-1 29380221 10.1007/s11060-018-2748-1

[41] Phonwijit L, Khawprapa C, Sitthinamsuwan B (2017) Progression-free survival and factors associated with postoperative recurrence in 126 patients with atypical intracranial meningioma. World Neurosurg 107:698–705. 10.1016/j.wneu.2017.08.057 28838877 10.1016/j.wneu.2017.08.057

[42] Park CJ, Choi SH, Eom J, Byun HK, Ahn SS, Chang JH, Kim SH, Lee SK, Park YW, Yoon HI (2022) An interpretable radiomics model to select patients for radiotherapy after surgery for WHO grade 2 meningiomas. Radiat Oncol 17:147. 10.1186/s13014-022-02090-7 35996160 10.1186/s13014-022-02090-7 PMC9396861

[43] Nowak A, Dziedzic T, Krych P, Czernicki T, Kunert P, Marchel A (2015) Benign versus atypical meningiomas: risk factors predicting recurrence. Neurol Neurochir Pol 49:1–10. 10.1016/j.pjnns.2014.11.003 25666766 10.1016/j.pjnns.2014.11.003

[44] Endo T, Narisawa A, Ali HSM, Murakami K, Watanabe T, Watanabe M, Jokura H, Endo H, Fujimura M, Sonoda Y, Tominaga T (2016) A study of prognostic factors in 45 cases of atypical meningioma. Acta Neurochir (Wien) 158:1661–1667. 10.1007/s00701-016-2900-7 27468919 10.1007/s00701-016-2900-7

[45] Choi Y, Lim DH, Yu JI, Jo K, Nam DH, Seol HJ, Lee JI, Kong DS, Suh YL, Nam H (2018) Prognostic value of Ki-67 labeling index and postoperative radiotherapy in WHO grade II meningioma. Am J Clin Oncol 41:18–23. 10.1097/COC.0000000000000224 26270441 10.1097/COC.0000000000000224

[46] Lee SH, Lee EH, Sung KS, Kim DC, Kim YZ, Song YJ (2022) Ki67 index is the most powerful factor for predicting the recurrence in atypical meningioma: retrospective analysis of 99 patients in two institutes. J Korean Neurosurg Soc 65:558–571. 10.3340/jkns.2021.0196 35418005 10.3340/jkns.2021.0196 PMC9271814

[47] Lee KD, DePowell JJ, Air EL, Dwivedi AK, Kendler A, McPherson CM (2013) Atypical meningiomas: is postoperative radiotherapy indicated? Neurosurg Focus 35:E15. 10.3171/2013.9.FOCUS13325 24289123 10.3171/2013.9.FOCUS13325

[48] Chang WI, Byun HK, Lee JH, Park CK, Kim IA, Kim CY, Chang JH, Kang SG, Lee SH, Kuranari Y, Tamura R, Toda M, Wee CW, Yoon HI (2023) Novel postoperative serum biomarkers in atypical meningiomas: a multicenter study. Neurosurgery 93:599–610. 10.1227/neu.0000000000002457 36921247 10.1227/neu.0000000000002457 PMC10827320

[49] Poulen G, Vignes JR, Le Corre M, Loiseau H, Bauchet L (2020) WHO grade II meningioma: epidemiology, survival and contribution of postoperative radiotherapy in a multicenter cohort of 88 patients. Neurochirurgie 66:73–79. 10.1016/j.neuchi.2019.12.008 32145249 10.1016/j.neuchi.2019.12.008

[50] Goyal LK, Suh JH, Mohan DS, Prayson RA, Lee J, Barnett GH (2000) Local control and overall survival in atypical meningioma: a retrospective study. Int J Radiat Oncol Biol Phys 46:57–61. 10.1016/s0360-3016(99)00349-1 10656373 10.1016/s0360-3016(99)00349-1

[51] Sahm F, Schrimpf D, Stichel D, Jones DTW, Hielscher T, Schefzyk S, Okonechnikov K, Koelsche C, Reuss DE, Capper D, Sturm D, Wirsching H-G, Berghoff AS, Baumgarten P, Kratz A, Huang K, Wefers AK, Hovestadt V, Sill M, Ellis HP, Kurian KM, Okuducu AF, Jungk C, Drueschler K, Schick M, Bewerunge-Hudler M, Mawrin C, Seiz-Rosenhagen M, Ketter R, Simon M, Westphal M, Lamszus K, Becker A, Koch A, Schittenhelm J, Rushing EJ, Collins VP, Brehmer S, Chavez L, Platten M, Hänggi D, Unterberg A, Paulus W, Wick W, Pfister SM, Mittelbronn M, Preusser M, Herold-Mende C, Weller M, von Deimling A (2017) DNA methylation-based classification and grading system for meningioma: a multicentre, retrospective analysis. Lancet Oncol 18:682–694. 10.1016/S1470-2045(17)30155-9 28314689 10.1016/S1470-2045(17)30155-9

[52] Nassiri F, Liu J, Patil V, Mamatjan Y, Wang JZ, Hugh-White R, Macklin AM, Khan S, Singh O, Karimi S, Corona RI, Liu LY, Chen CY, Chakravarthy A, Wei Q, Mehani B, Suppiah S, Gao A, Workewych AM, Tabatabai G, Boutros PC, Bader GD, De Carvalho DD, Kislinger T, Aldape K, Zadeh G (2021) A clinically applicable integrative molecular classification of meningiomas. Nature 597:119–125. 10.1038/s41586-021-03850-3 34433969 10.1038/s41586-021-03850-3 PMC11604310

[53] Driver J, Hoffman SE, Tavakol S, Woodward E, Maury EA, Bhave V, Greenwald NF, Nassiri F, Aldape K, Zadeh G, Choudhury A, Vasudevan HN, Magill ST, Raleigh DR, Abedalthagafi M, Aizer AA, Alexander BM, Ligon KL, Reardon DA, Wen PY, Al-Mefty O, Ligon AH, Dubuc AM, Beroukhim R, Claus EB, Dunn IF, Santagata S, Bi WL (2022) A molecularly integrated grade for meningioma. Neuro Oncol 24:796–808. 10.1093/neuonc/noab213 34508644 10.1093/neuonc/noab213 PMC9071299

[54] Hanakita S, Koga T, Igaki H, Murakami N, Oya S, Shin M, Saito N (2013) Role of Gamma Knife surgery for intracranial atypical (WHO Grade II) meningiomas: clinical article. J Neurosurg 119:1410–1414. 10.3171/2013.8.JNS13343 24074490 10.3171/2013.8.JNS13343

[55] Harris AE, Lee JYK, Omalu B, Flickinger JC, Kondziolka D, Lunsford LD (2003) The effect of radiosurgery during management of aggressive meningiomas. Surg Neurol 60:298–305. 10.1016/S0090-3019(03)00320-3 14505844 10.1016/s0090-3019(03)00320-3

[56] Chen WC, Perlow HK, Choudhury A, Nguyen MP, Mirchia K, Youngblood MW, Lucas C-HG, Palmer JD, Magill ST, Raleigh DR (2022) Radiotherapy for meningiomas. J Neurooncol 160:505–515. 10.1007/s11060-022-04171-9 36315366 10.1007/s11060-022-04171-9 PMC9722800

[57] Goldbrunner R, Stavrinou P, Jenkinson MD, Sahm F, Mawrin C, Weber DC, Preusser M, Minniti G, Lund-Johansen M, Lefranc F, Houdart E, Sallabanda K, Le Rhun E, Nieuwenhuizen D, Tabatabai G, Soffietti R, Weller M (2021) EANO guideline on the diagnosis and management of meningiomas. Neuro Oncol 23:1821–1834. 10.1093/neuonc/noab150 34181733 10.1093/neuonc/noab150 PMC8563316

[58] Jenkinson MD, Javadpour M, Haylock BJ, Young B, Gillard H, Vinten J, Bulbeck H, Das K, Farrell M, Looby S, Hickey H, Preusser M, Mallucci CL, Hughes D, Gamble C, Weber DC (2015) The ROAM/EORTC-1308 trial: radiation versus observation following surgical resection of atypical meningioma: study protocol for a randomised controlled trial. Trials 16:519. 10.1186/s13063-015-1040-3 26576533 10.1186/s13063-015-1040-3 PMC4650615

[59] Abualnaja SY, Morris JS, Rashid H, Cook WH, Helmy AE (2024) Machine learning for predicting post-operative outcomes in meningiomas: a systematic review and meta-analysis. Acta Neurochir (Wien) 166:505. 10.1007/s00701-024-06344-z 39688716 10.1007/s00701-024-06344-z PMC11652405

[60] Cook WH, Khalil F, Gillespie CS, Helmy AE (2025) Health-related quality-of-life outcomes in CNS WHO grade 2 and 3 meningioma: a systematic review. Neurosurg Rev 48:268. 10.1007/s10143-025-03420-5 40011234 10.1007/s10143-025-03420-5 PMC11865157

[61] Keshwara SM, Gillespie CS, Mustafa MA, George AM, Richardson GE, Clynch AL, Wang JZ, Lawson DDA, Gilkes CE, Farah JO, Yousaf J, Chavredakis E, Mills SJ, Brodbelt AR, Zadeh G, Millward CP, Islim AI, Jenkinson MD (2023) Quality of life outcomes in incidental and operated meningiomas (QUALMS): a cross-sectional cohort study. J Neurooncol 161:317–327. 10.1007/s11060-022-04198-y 36525165 10.1007/s11060-022-04198-y PMC9756745

[62] Zamanipoor Najafabadi AH, Dirven L, Drummond KJ, Taphoorn MJB (2023) Health-Related Quality of Life in Intracranial Meningioma: Current Evidence and Future Directions. In: Zadeh G, Goldbrunner R, Krischek B, Nassiri F (eds) Biological and Clinical Landscape of Meningiomas. Springer International Publishing, Cham, pp 235–252 10.1007/978-3-031-29750-2_18 37432632

[63] Millward CP, Armstrong TS, Barrington H, Bell S, Brodbelt AR, Bulbeck H, Crofton A, Dirven L, Georgious T, Grundy PL, Islim AI, Javadpour M, Keshwara SM, Koszdin SD, Marson AG, McDermott MW, Meling TR, Oliver K, Plaha P, Preusser M, Santarius T, Srikandarajah N, Taphoorn MJB, Turner C, Watts C, Weller M, Williamson PR, Zadeh G, Zamanipoor Najafabadi AH, Jenkinson MD (2022) Development of ‘core outcome sets’ for meningioma in clinical studies (the COSMIC Project): protocol for two systematic literature reviews, eDelphi surveys and online consensus meetings. BMJ Open 12:e057384. 10.1136/bmjopen-2021-057384 10.1136/bmjopen-2021-057384 PMC9086638 35534067

