# Supplementary material for: Postoperative outcomes in CNS WHO grade 2 and 3 meningioma: a systematic review and meta-analysis
Source: Langenbecks Arch Surg. 2026 May 18;411(1):189. doi: 10.1007/s00423-026-04081-8 (PMC13350211; doi:10.1007/s00423-026-04081-8)
Supplement: Supplementary file 1 — Supplementary Material 1 [file 423_2026_4081_MOESM1_ESM.docx]

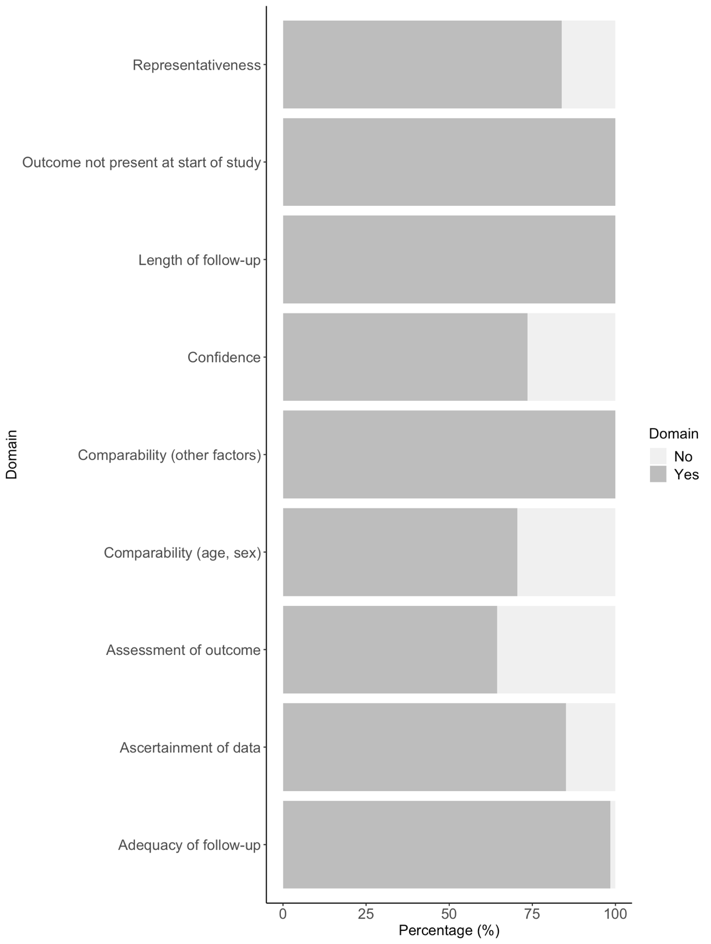


**Supplemental Figure 1.** Stacked bar chart of risk of bias for observational studies (N=74) using the Newcastle-Ottawa Scale.


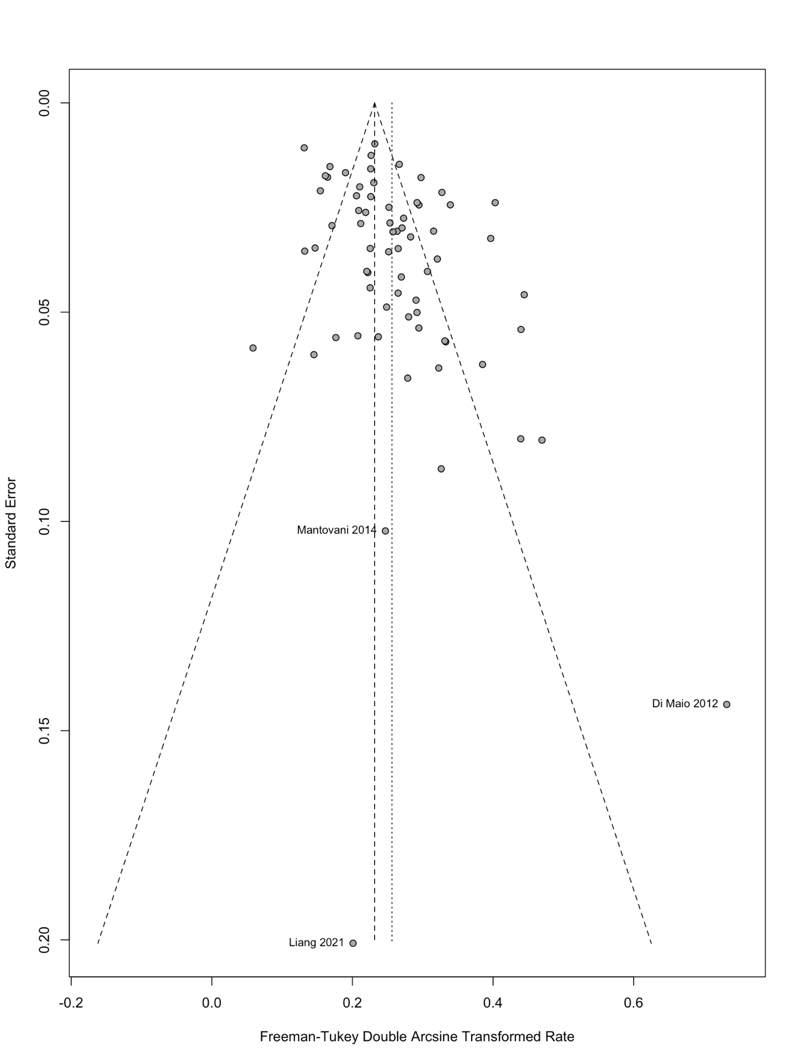


**Supplemental Figure 2.** Funnel plot of annual incidence of recurrence in CNS WHO grade 2 meningioma. Selected outlying studies labelled.
