# Supplementary material for: Postoperative outcomes in CNS WHO grade 2 and 3 meningioma: a systematic review and meta-analysis
Source: Langenbecks Arch Surg. 2026 May 18;411(1):189. doi: 10.1007/s00423-026-04081-8 (PMC13350211; doi:10.1007/s00423-026-04081-8)
Supplement: Supplementary file 3 — Supplementary Material 3 [file 423_2026_4081_MOESM3_ESM.docx]

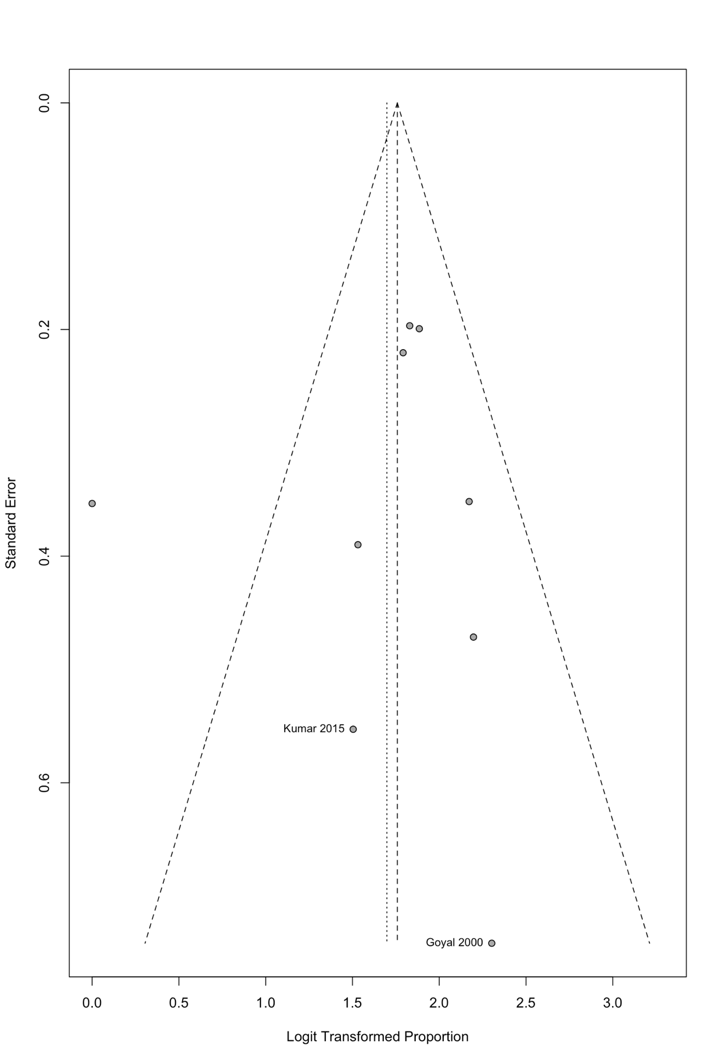


**Supplemental Figure 5.** Funnel plot of 5-year OS in CNS WHO grade 2 meningioma. Selected outlying studies labelled.


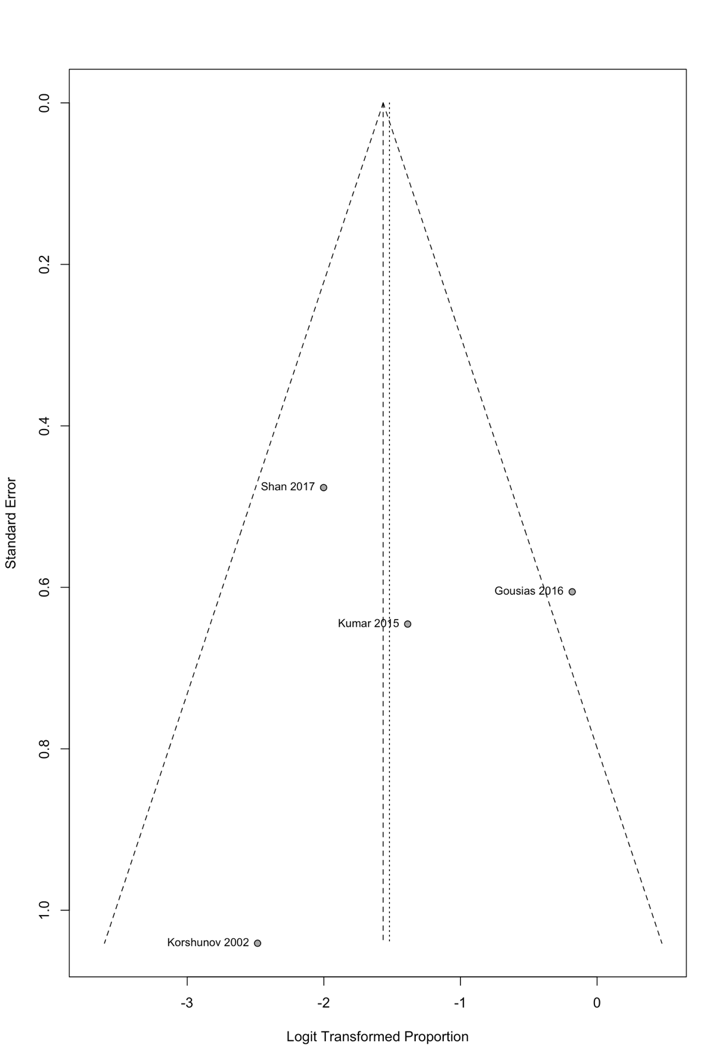


**Supplemental Figure 6.** Funnel plot of 5-year PFS in CNS WHO grade 3 meningioma.


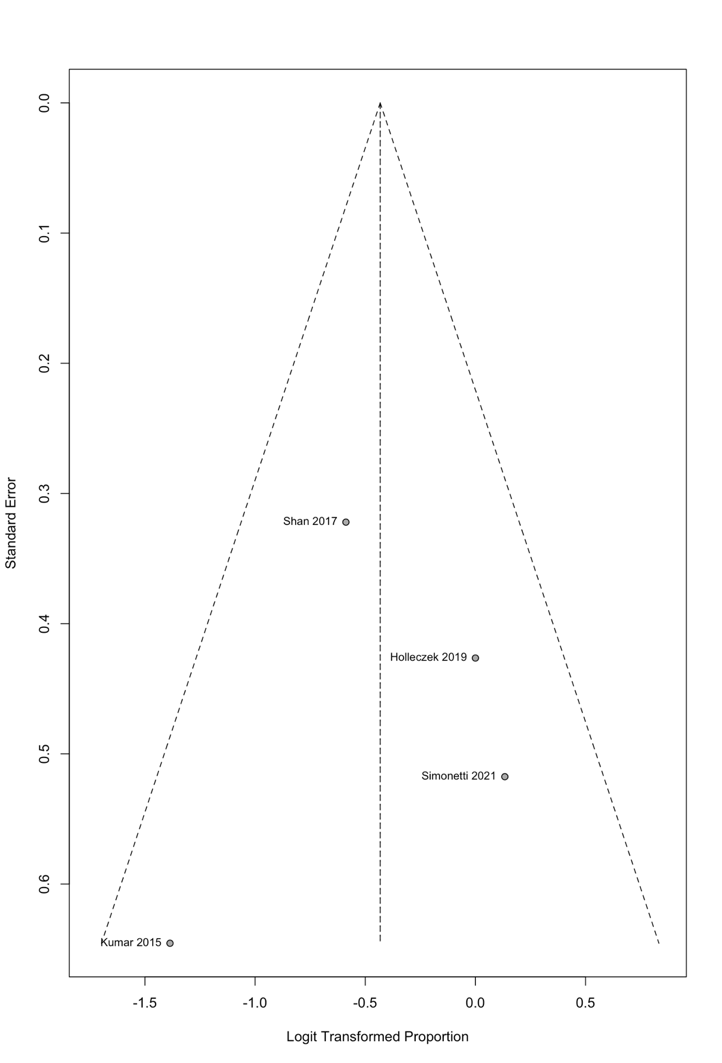


**Supplemental Figure 7.** Funnel plot of 5-year OS in CNS WHO grade 3 meningioma.
