# Supplementary material for: Postoperative outcomes in CNS WHO grade 2 and 3 meningioma: a systematic review and meta-analysis
Source: Langenbecks Arch Surg. 2026 May 18;411(1):189. doi: 10.1007/s00423-026-04081-8 (PMC13350211; doi:10.1007/s00423-026-04081-8)
Supplement: Supplementary file 4 — Supplementary Material 4 [file 423_2026_4081_MOESM4_ESM.docx]

**Supplemental Digital Content**

**Appendix 1.** Ovid MEDLINE Search

1 (atypical adj6 meningioma*).mp. [mp=title, book title, abstract, original title, name of substance word, subject heading word, floating sub-heading word, keyword heading word, organism supplementary concept word, protocol supplementary concept word, rare disease supplementary concept word, unique identifier, synonyms, population supplementary concept word, anatomy supplementary concept word] 1136

2 (malignant adj6 meningioma*).mp. [mp=title, book title, abstract, original title, name of substance word, subject heading word, floating sub-heading word, keyword heading word, organism supplementary concept word, protocol supplementary concept word, rare disease supplementary concept word, unique identifier, synonyms, population supplementary concept word, anatomy supplementary concept word] 1168

3 (grade adj2 II adj6 meningioma*).mp. [mp=title, book title, abstract, original title, name of substance word, subject heading word, floating sub-heading word, keyword heading word, organism supplementary concept word, protocol supplementary concept word, rare disease supplementary concept word, unique identifier, synonyms, population supplementary concept word, anatomy supplementary concept word] 583

4 (grade adj2 III adj6 meningioma*).mp. [mp=title, book title, abstract, original title, name of substance word, subject heading word, floating sub-heading word, keyword heading word, organism supplementary concept word, protocol supplementary concept word, rare disease supplementary concept word, unique identifier, synonyms, population supplementary concept word, anatomy supplementary concept word] 334

5 (grade adj2 "2" adj6 meningioma*).mp. 120

6 (grade adj2 "3" adj6 meningioma*).mp. [mp=title, book title, abstract, original title, name of substance word, subject heading word, floating sub-heading word, keyword heading word, organism supplementary concept word, protocol supplementary concept word, rare disease supplementary concept word, unique identifier, synonyms, population supplementary concept word, anatomy supplementary concept word] 55

7 exp Specialties, Surgical/ 218776

8 surg*.mp. [mp=title, book title, abstract, original title, name of substance word, subject heading word, floating sub-heading word, keyword heading word, organism supplementary concept word, protocol supplementary concept word, rare disease supplementary concept word, unique identifier, synonyms, population supplementary concept word, anatomy supplementary concept word] 3307403

9 neurosurg*.mp. [mp=title, book title, abstract, original title, name of substance word, subject heading word, floating sub-heading word, keyword heading word, organism supplementary concept word, protocol supplementary concept word, rare disease supplementary concept word, unique identifier, synonyms, population supplementary concept word, anatomy supplementary concept word] 83109

10 exp Radiotherapy/ 206643

11 radiotherap*.mp. [mp=title, book title, abstract, original title, name of substance word, subject heading word, floating sub-heading word, keyword heading word, organism supplementary concept word, protocol supplementary concept word, rare disease supplementary concept word, unique identifier, synonyms, population supplementary concept word, anatomy supplementary concept word] 342743

12 radiosurg*.mp. [mp=title, book title, abstract, original title, name of substance word, subject heading word, floating sub-heading word, keyword heading word, organism supplementary concept word, protocol supplementary concept word, rare disease supplementary concept word, unique identifier, synonyms, population supplementary concept word, anatomy supplementary concept word] 22720

13 Radiation therap*.mp. [mp=title, book title, abstract, original title, name of substance word, subject heading word, floating sub-heading word, keyword heading word, organism supplementary concept word, protocol supplementary concept word, rare disease supplementary concept word, unique identifier, synonyms, population supplementary concept word, anatomy supplementary concept word] 78696

14 exp Drug Therapy/ 1504164

15 chemotherap*.mp. [mp=title, book title, abstract, original title, name of substance word, subject heading word, floating sub-heading word, keyword heading word, organism supplementary concept word, protocol supplementary concept word, rare disease supplementary concept word, unique identifier, synonyms, population supplementary concept word, anatomy supplementary concept word] 509484

16 1 or 2 or 3 or 4 or 5 or 6 2499

17 8 or 9 or 10 or 11 or 12 or 13 or 14 or 15 5036580

18 16 and 17 1479

EMBASE Search

1. (atypical adj6 meningioma*).mp. [mp=title, book title, abstract, original title, name of substance word, subject heading word, floating sub-heading word, keyword heading word, organism supplementary concept word, protocol supplementary concept word, rare disease supplementary concept word, unique identifier, synonyms, population supplementary concept word, anatomy supplementary concept word]

2. (malignant adj6 meningioma*).mp. [mp=title, book title, abstract, original title, name of substance word, subject heading word, floating sub-heading word, keyword heading word, organism supplementary concept word, protocol supplementary concept word, rare disease supplementary concept word, unique identifier, synonyms, population supplementary concept word, anatomy supplementary concept word]

3. (grade adj2 II adj6 meningioma*).mp. [mp=title, book title, abstract, original title, name of substance word, subject heading word, floating sub-heading word, keyword heading word, organism supplementary concept word, protocol supplementary concept word, rare disease supplementary concept word, unique identifier, synonyms, population supplementary concept word, anatomy supplementary concept word]

4. (grade adj2 III adj6 meningioma*).mp. [mp=title, book title, abstract, original title, name of substance word, subject heading word, floating sub-heading word, keyword heading word, organism supplementary concept word, protocol supplementary concept word, rare disease supplementary concept word, unique identifier, synonyms, population supplementary concept word, anatomy supplementary concept word]

5. (grade adj2 "2" adj6 meningioma*).mp.

6. (grade adj2 "3" adj6 meningioma*).mp. [mp=title, book title, abstract, original title, name of substance word, subject heading word, floating sub-heading word, keyword heading word, organism supplementary concept word, protocol supplementary concept word, rare disease supplementary concept word, unique identifier, synonyms, population supplementary concept word, anatomy supplementary concept word]

7. exp Specialties, Surgical/

8. surg*.mp. [mp=title, book title, abstract, original title, name of substance word, subject heading word, floating sub-heading word, keyword heading word, organism supplementary concept word, protocol supplementary concept word, rare disease supplementary concept word, unique identifier, synonyms, population supplementary concept word, anatomy supplementary concept word]

9. neurosurg*.mp. [mp=title, book title, abstract, original title, name of substance word, subject heading word, floating sub-heading word, keyword heading word, organism supplementary concept word, protocol supplementary concept word, rare disease supplementary concept word, unique identifier, synonyms, population supplementary concept word, anatomy supplementary concept word]

10. exp Radiotherapy/

11. radiotherap*.mp. [mp=title, book title, abstract, original title, name of substance word, subject heading word, floating sub-heading word, keyword heading word, organism supplementary concept word, protocol supplementary concept word, rare disease supplementary concept word, unique identifier, synonyms, population supplementary concept word, anatomy supplementary concept word]

12. radiosurg*.mp. [mp=title, book title, abstract, original title, name of substance word, subject heading word, floating sub-heading word, keyword heading word, organism supplementary concept word, protocol supplementary concept word, rare disease supplementary concept word, unique identifier, synonyms, population supplementary concept word, anatomy supplementary concept word]

13. Radiation therap*.mp. [mp=title, book title, abstract, original title, name of substance word, subject heading word, floating sub-heading word, keyword heading word, organism supplementary concept word, protocol supplementary concept word, rare disease supplementary concept word, unique identifier, synonyms, population supplementary concept word, anatomy supplementary concept word]

14. exp Drug Therapy/

15. chemotherap*.mp. [mp=title, book title, abstract, original title, name of substance word, subject heading word, floating sub-heading word, keyword heading word, organism supplementary concept word, protocol supplementary concept word, rare disease supplementary concept word, unique identifier, synonyms, population supplementary concept word, anatomy supplementary concept word]

16. 1 or 2 or 3 or 4 or 5 or 6

17. 8 or 9 or 10 or 11 or 12 or 13 or 14 or 15

18. 16 and 17

Cochrane Library Search

ID Search

#1 atypical NEAR meningioma*

#2 malignant NEAR meningioma*

#3 grade NEAR/2 II NEAR meningioma*

#4 grade NEAR/2 III NEAR meningioma*

#5 grade NEAR/2 "2" NEAR meningioma*

#6 grade NEAR/2 "3" NEAR meningioma*

#7 MeSH descriptor: [Specialties, Surgical] explode all trees

#8 surg*

#9 neurosurg*

#10 MeSH descriptor: [Radiotherapy] explode all trees

#11 radiotherap*

#12 radiosurg*

#13 radiation therap*

#14 MeSH descriptor: [Drug Therapy] explode all trees

#15 chemotherap*

#16 #1 OR #2 OR #3 OR #4 OR #5 OR #6

#17 #7 OR #8 OR #9 OR #10 OR #11 OR #12 OR #13 OR #14 OR #15

#18 #16 AND #17

| **Supplemental Table 1.** Baseline demographics, recurrence rates, and 5-year PFS and OS rates for studies of CNS WHO grade 2 meningioma. | | | | | | |
| --- | --- | --- | --- | --- | --- | --- |
| **Author and year** | **Sample size** | **Average follow-up time (years)** | **Definitions** | **Annual incidence of recurrence** | **Average 5-year PFS** | **Average 5-year OS** |
| **Prospective** | | | | | | |
| Weber et al., 2018^1^ | 56 | 5.10 | Recurrence: MRI new lesions or increase in tumour size by 25% | 0.03 |  |  |
| **Retrospective** | | | | | | |
| Tena-Suck et al., 2010^2^ | 10 | 3.88 | Recurrence: none | 0.18 |  |  |
| Yu et al., 2020^3^ | 21 | 5.00 | Recurrence: none | 0.08 |  |  |
| Aboukais et al., 2013^4^ | 167 | 4.7 | Recurrence: none | 0.09 |  |  |
| Anand et al., 2021^5^ | 103 | 4.08 | Recurrence: none | 0.09 |  |  |
| Barresi et al., 2023^6^ | 55 | 4.33 | Recurrence: none | 0.16 |  |  |
| Yip et al., 2016^7^ | 27 | 4.17 | Recurrence: MRI re-growth or progression | 0.08 |  |  |
| Behling et al., 2020^8^ | 232 | 5.00 | Recurrence: none | 0.13 |  |  |
| Budohoski et al., 2018^9^ | 220 | 2.00 | Recurrence: presence of tumour where there was no tumour on post-operative MRI  PFS: none  OS: none | 0.16 | 0.59 | 0.87 |
| Celtikci et al., 2018^10^ | 112 | 4.45 | Recurrence: none | 0.05 |  |  |
| Unterberger et al., 2021^11^ | 43 | 3.58 | Recurrence: new lesion of any size on MRI or CT after GTR, any growth after STR  PFS: surgery🡪local failure | 0.09 | 0.53 |  |
| Ukai et al., 2020^12^ | 28 | 9.50 | Recurrence: radiological | 0.07 |  |  |
| Turner et al., 2022^13^ | 119 | 6.67 | Recurrence: radiological | 0.03 |  |  |
| Torres-Bayona et al., 2021^14^ | 32 | 2.67 | Recurrence: none  PFS: surgery🡪recurrence/progression  OS: surgery🡪death/last follow-up | 0.19 | 0.56 | 0.50 |
| Devalckeneer et al., 2022^15^ | 9 | 8.10 | Recurrence: MRI | 0.00 |  |  |
| Simonetti et al., 2021^16^ | 168 | 3.70 | Recurrence: none  OS: surgery🡪death | 0.04 |  | 0.86 |
| Sekerci et al., 2004^17^ | 26 | 8.00 | Recurrence: none | 0.02 |  |  |
| Sadashiva et al., 2018^18^ | 33 | 4.61 | Recurrence: none  PFS: none | 0.05 | 0.88 |  |
| Ros-Sanjuan et al., 2019^19^ | 28 | 6.42 | Recurrence: ‘regular control imaging tests’ | 0.10 |  |  |
| Rebchuk et al., 2022^20^ | 189 | 5.33 | Recurrence: serial imaging | 0.05 |  |  |
| Press et al., 2014^21^ | 46 | 2.17 | Recurrence: serial brain MRI with contrast | 0.08 |  |  |
| Prat-Acin et al., 2021^22^ | 43 | 4.81 | Recurrence: annual MRI | 0.05 |  |  |
| Poulen et al., 2020^23^ | 88 | 5.77 | Recurrence: ‘regular MRI’  OS: time from first  surgery (corresponding to histological diagnosis) to death or else  censored at the date of last MRI follow-up. | 0.04 |  | 0.90 |
| Piscevic et al., 2015^24^ | 75 | 5.62 | Recurrence: presence of new pathologic tissue on repeated postoperative imaging (not noticed at an earlier control) or further growth of pathologic tissue already detected on the previous postoperative CT or MRI. | 0.11 |  |  |
| Phonwijit et al., 2017^25^ | 126 | 4.33 | Recurrence: either contrast- enhanced cranial CT or MRI or both.  PFS: none | 0.11 | 0.72 |  |
| Pessina et al., 2019^26^ | 61 | 6.58 | Recurrence: tumor growth resulting in symptomatic changes was considered a recurrence. In asymptomatic cases, tumor recurrence was defined as ≥10% of growth in diameter or volume. | 0.06 |  |  |
| Park et al., 2019a^27^ | 13 | 5.32 | Recurrence: No change in tumor size or no evidence of recurrence in follow-up MRI was defined as “stable disease”; “tumor progression” covered all other circumstances | 0.02 |  |  |
| Park et al., 2019b^28^ | 20 | 3.12 | Recurrence: increase in lesion size after GKS or development of a new lesion around the previously irradiated lesion on follow-up MRI | 0.10 |  |  |
| Park et al., 2022^29^ | 155 | 5.32 | Recurrence: none  PFS: time from initial surgery to tumor recurrence, death, or the last follow-up | 0.03 | 0.79 |  |
| Moon et al., 2012^30^ | 55 | 3.75 | Recurrence: none | 0.07 |  |  |
| Momin et al., 2022^31^ | 160 | 2.75 | Recurrence: radiographic evidence of tumour recurrence by analysis of both imaging and clinical reports. | 0.11 |  |  |
| Mattozo et al., 2007^32^ | 11 | 3.50 | Recurrence: MRI | 0.21 |  |  |
| Mantovani et al., 2014^33^ | 11 | 2.17 | Recurrence: none | 0.04 |  |  |
| Liang et al., 2021^34^ | 6 | 1.03 | Recurrence: none | 0.00 |  |  |
| Lee et al., 2021^35^ | 230 | 6.90 | Recurrence: imaging | 0.05 |  |  |
| Di Ieva et al., 2015^36^ | 8 | 4.09 | Recurrence: none | 0.09 |  |  |
| Kumar et al., 2015^37^ | 22 | 3.67 | Recurrence: clinical evaluation and imaging  PFS: none  OS: none | 0.04 | 0.59 | 0.82 |
| Korshunov et al., 2002^38^ | 42 | 2.83 | Recurrence: CT or MRI  PFS: none | 0.19 | 0.43 |  |
| Ko et al., 2007^39^ | 31 | 3.08 | Recurrence: radiographic | 0.07 |  |  |
| Klinger et al., 2015^40^ | 45 | 5.42 | Recurrence: none | 0.08 |  |  |
| Kim et al., 2014^41^ | 67 | 3.98 | Recurrence: new tumor in patients with a completely resected tumour, as judged on the first postoperative MRI, or as evidence of new growth of an incompletely resected tumour on serial postoperative MRI compared with the immediate postoperative MRI. | 0.10 |  |  |
| Morokoff et al., 2008^42^ | 16 | 5.00 | Recurrence: new enhancing tumor on the computed tomographic scan or magnetic resonance imaging (MRI) scan or, in the absence of imaging data, a second procedure (surgery or radiotherapy) for the same tumour location.  PFS: none | 0.05 | 0.56 |  |
| Nowak et al., 2014^43^ | 44 | 6.83 | Recurrence: MRI  PFS: date of surgery to  the date of recurrence | 0.04 | 0.70 |  |
| Karabagli et al., 2020^44^ | 75 | 3.73 | Recurrence: Contrast-enhanced T1-weighted MRI | 0.07 |  |  |
| Kalasauskas et al., 2020^45^ | 76 | 3.47 | Recurrence: none | 0.06 |  |  |
| Joseph et al., 2000^46^ | 32 | 2.00 | Recurrence: none | 0.14 |  |  |
| James et al., 2023^47^ | 18 | 4.42 | Recurrence: none | 0.03 |  |  |
| Holleczek et al., 2019^48^ | 217 | 10.00 | Recurrence: none | 0.02 |  | 0.86 |
| Hammouche et al., 2014^49^ | 79 | 4.17 | Recurrence: none | 0.07 |  |  |
| Halliday et al., 2010^50^ | 42 | 9.00 | Recurrence: radiological change, on CT or MRI scanning, relative to previous scans performed | 0.02 |  |  |
| Gurses et al., 2023^51^ | 23 | 3.33 | Recurrence: radiological evidence of tumour regrowth after surgery | 0.10 |  |  |
| Goyal et al., 2000^52^ | 22 | 5.50 | Recurrence: imaging  OS: none | 0.07 |  | 0.91 |
| Gousias et al., 2016^53^ | 174 | 5.17 | Recurrence: MRI  PFS: none | 0.00 | 0.82 |  |
| Fernandez et al., 2016^54^ | 50 | 3.08 | Recurrence: imaging | 0.05 |  |  |
| Escribano Mesa et al., 2018^55^ | 16 | 8.00 | Recurrence: none | 0.05 |  |  |
| Endo et al., 2016^56^ | 45 | 6.75 | Recurrence:  PFS: recurrence date when second surgery performed or when radiotherapy applied for imaging recurrence  OS: surgery🡪death | 0.06 | 0.58 | 0.82 |
| Di Maio et al., 2012^57^ | 6 | 2.02 | Recurrence: none | 0.5 |  |  |
| Choi et al., 2018^58^ | 50 | 3.95 | Recurrence: imaging  PFS: surgery🡪local failure and last follow-up  OS: surgery🡪death/last follow-up | 0.06 | 0.66 | 0.90 |
| Barresi et al., 2007^59^ | 26 | 7.67 | Recurrence: imaging | 0.06 |  |  |
| Andric et al., 2012^60^ | 9 | 8.58 | Recurrence: none | 0.10 |  |  |
| Aghi et al., 2009^61^ | 108 | 10.00 | Recurrence: none | 0.05 |  |  |
| Aboukais et al., 2015^62^ | 9 | 6.42 | Recurrence: MRI | 0.07 |  |  |
| Lee et al., 2022b^63^ | 99 | 6.94 | Recurrence: none | 0.05 | 0.75 |  |
| Lee et al., 2013^64^ | 90 | 4.06 | Recurrence: none | 0.05 | 0.81 |  |
| Unteroberdorster et al., 2021^65^ | 28 | 5.16 | Recurrence: none | 0.07 |  |  |
| Chang et al., 2023^66^ | 523 | 5.00 | Recurrence: imaging  PFS: surgery🡪recurrence/death | 0.05 | 0.72 |  |
| Torp et al., 2005^67^ | 17 | 5.08 | Recurrence: radiology, reoperation, death caused by tumour | 0.08 |  |  |
| Sun et al., 2014^68^ | 151 | 3.75 | Recurrence: MRI | 0.02 |  |  |
| PFS, progression-free survival; OS, overall survival. | | | | | | |

| **Supplemental Table 2.** Baseline demographics, recurrence rates, and 5-year PFS and OS rates for studies of CNS WHO grade 3 meningioma. | | | | | | |
| --- | --- | --- | --- | --- | --- | --- |
| **Author and year** | **Sample size** | **Average follow-up time (years)** | **Definitions** | **Annual incidence of recurrence** | **Average 5-year PFS** | **Average 5-year OS** |
| **Prospective** | | | | | | |
| Weber et al., 2018^1^ | 9 | 5.1 | Recurrence: MRI new lesions or increase in tumour size by 25% | 0.04 |  |  |
| **Retrospective** | | | | | | |
| Shan et al., 2017^69^ | 42 | 1.93 | Recurrence: imaging  PFS: surgery🡪recurrence  OS: surgery🡪death (all causes) | 0.37 | 0.12 | 0.36 |
| Ramina et al., 2006^70^ | 5 | 1.28 | Recurrence: none | 0.47 |  |  |
| Kim et al., 2007^71^ | 15 | 1.93 | Recurrence: annual MRI | 0.03 |  |  |
| Kim et al., 2009^72^ | 4 | 1.46 | Recurrence: CT/MRI | 0.17 |  |  |
| Ildan et al., 2007^73^ | 2 | 5.67 | Recurrence: none | 0.18 |  |  |
| Li et al., 2016^74^ | 10 | 3.55 | Recurrence: MRI | 0.17 |  |  |
| Yu et al., 2020^3^ | 16 | 5.00 | Recurrence: none | 0.08 |  |  |
| Behling et al., 2020^8^ | 3 | 5.00 | Recurrence: none | 0.13 |  |  |
| Turner et al., 2022^13^ | 5 | 6.67 | Recurrence: imaging | 0.06 |  |  |
| Simonetti et al., 2021^16^ | 15 | 3.70 | Recurrence: none  OS: surgery🡪death | 0.05 |  | 0.50 |
| Sekerci et al., 2004^17^ | 19 | 9.00 | Recurrence: none | 0.04 |  |  |
| Piscevic et al., 2015^24^ | 13 | 5.60 | Recurrence: presence of new pathologic tissue on repeated postoperative imaging (not noticed at an earlier control) or further growth of pathologic tissue already detected on the previous postoperative CT or MRI. | 0.14 |  |  |
| Park et al., 2019b^28^ | 2 | 3.12 | Recurrence: increase in lesion size after GKS or development of a new lesion around the previously irradiated lesion on follow-up MRI | 0.16 |  |  |
| Kumar et al., 2015^37^ | 15 | 3.67 | Recurrence: clinical evaluation and imaging  PFS: none  OS: none | 0.18 | 0.20 | 0.23 |
| Korshunov et al., 2002^38^ | 13 | 2.83 | Recurrence: CT or MRI  PFS: none | 0.35 | 0.08 |  |
| Ko et al., 2007^39^ | 20 | 3.08 | Recurrence: radiographic | 0.11 |  |  |
| Morokoff et al., 2008^42^ | 3 | 5.00 | Recurrence: new enhancing tumor on the computed tomographic scan or magnetic resonance imaging (MRI) scan or, in the absence of imaging data, a second procedure (surgery or radiotherapy) for the same tumour location. | 0.13 |  |  |
| Holleczek et al., 2019^48^ | 22 | 10.00 | Recurrence: none | 0.03 |  | 0.50 |
| Halliday et al., 2010^50^ | 6 | 9.00 | Recurrence: radiological change, on CT or MRI scanning, relative to previous scans performed | 0.06 |  |  |
| Gousias et al., 2016^53^ | 11 | 5.17 | Recurrence: MRI  PFS: none | 0.05 | 0.47 |  |
| Escribano Mesa et al., 2018^55^ | 3 | 8.00 | Recurrence: none | 0.08 |  |  |
| PFS, progression-free survival; OS, overall survival. | | | | | | |

| **Supplemental Table 3.** Sensitivity analysis of annual incidence of recurrence, 5-year PFS, and 5-year OS, adjusting for studies at high risk of bias and removing studies that did not define recurrence criteria. | | |
| --- | --- | --- |
|  | **Included studies (number of patients)** | **Estimate (95% CI)** |
| **CNS WHO grade 2 meningioma** | | |
| **Annual incidence of recurrence** | | |
| All studies | 68 (4937) | 0.06 (0.05-0.07) |
| Low risk of bias | 50 (3801) | 0.06 (0.05-0.07) |
| High risk of bias | 18 (1136) | 0.07 (0.05-0.10) |
| Recurrence definition | 39 (3030) | 0.06 (0.05-0.07) |
| No recurrence definition | 29 (1907) | 0.06 (0.05-0.08) |
| **5-year PFS** | | |
| All studies | 15 (1698) | 0.69 (0.63-0.75) |
| Low risk of bias | 8 (1169) | 0.67 (0.58-0.74) |
| High risk of bias | 7 (529) | 0.72 (0.64-0.80) |
| Recurrence definition | 10 (1289) | 0.65 (0.58-0.72) |
| No recurrence definition | 5 (409) | 0.76 (0.69-0.83) |
| **5-year OS** | | |
| All studies | 9 (864) | 0.85 (0.78-0.89) |
| Low risk of bias | 6 (614) | 0.87 (0.84-0.89) |
| High risk of bias | 3 (250) | 0.79 (0.57-0.92) |
| Recurrence definition | 6 (447) | 0.87 (0.84-0.90) |
| No recurrence definition | 3 (417) | 0.78 (0.58-0.90) |
| **CNS WHO grade 3 meningioma** | | |
| **Annual incidence of recurrence** | | |
| All studies | 22 (253) | 0.10 (0.07-0.14) |
| Low risk of bias | 20 (233) | 0.10 (0.07-0.14) |
| High risk of bias | 2 (20) | 0.10 (0.04-0.22) |
| Recurrence definition | 14 (168) | 0.12 (0.08-0.19) |
| No recurrence definition | 8 (85) | 0.07 (0.04-0.12) |
| **5-year PFS** | | |
| All studies | 4 (81) | 0.18 (0.09-0.33) |
| Low risk of bias | 4 (81) | 0.18 (0.09-0.33) |
| High risk of bias | 0 (0) | N/A |
| Recurrence definition | 4 (81) | 0.18 (0.09-0.33) |
| No recurrence definition | 0 (0) | N/A |
| **5-year OS** | | |
| All studies | 4 (94) | 0.39 (0.30-0.50) |
| Low risk of bias | 3 (79) | 0.37 (0.27-0.48) |
| High risk of bias | 1 (15) | N/A |
| Recurrence definition | 2 (57) | 0.32 (0.21-0.45) |
| No recurrence definition | 2 (37) | 0.51 (0.36-0.67) |
| *High risk of bias: ‘high’ risk of bias according to Newcastle Ottawa Scale. N/A, not applicable.  PFS, progression-free survival; OS, overall survival. | | |

**Supplemental Digital Content References**

1. Weber DC, Ares C, Villa S, et al. Adjuvant postoperative high-dose radiotherapy for atypical and malignant meningioma: a phase-II parallel non-randomized and observation study (EORTC 22042-26042). *Radiother Oncol*. 2018;128(2):260–265.

2. Tena-Suck ML, Collado-Ortiz MA, Salinas-Lara C, Garcia-Lopez R, Gelista N, Rembao-Bojorquez D. Chordoid meningioma: a report of ten cases. *J Neurooncol*. 2010;99(1):41–48.

3. Yu J, Chen FF, Zhang HW, et al. Comparative Analysis of the MRI Characteristics of Meningiomas According to the 2016 WHO Pathological Classification. *Technol Cancer Res Treat*. 2020;19

4. Aboukais R, Baroncini M, Zairi F, Reyns N, Lejeune JP. Early postoperative radiotherapy improves progression free survival in patients with grade 2 meningioma. *Acta Neurochir (Wien)*. 2013;155(8):1385–1390.

5. Anand SK, Sardari H, Sadeghsalehi A, et al. Radiopathologic predictors of recurrence in patients with a gross totally resected atypical meningioma. *Neurol Res*. 2021;44(5):468–474.

6. Barresi V, Ammendola S, Simbolo M, Pedron S, Caffo M, Scarpa A. Atypical meningiomas with an immunohistochemical profile consistent with hypermetabolic or proliferative molecular groups show high mitotic index, chromosomal instability, and higher recurrence risk. *Virchows Arch*. 2023;483(1):97–104.

7. Yip CM, Hsu SS, Liao WC, et al. Intracranial Atypical Meningiomas: A Case Series. *J Med Sci*. 2016;36(3):108–112.

8. Behling F, Fodi C, Gepfner-Tuma I, et al. CNS Invasion in Meningioma—How the Intraoperative Assessment Can Improve the Prognostic Evaluation of Tumor Recurrence. *Cancers (Basel)*. 2020;12(12):3620.

9. Budohoski KP, Clerkin J, Millward CP, et al. Predictors of early progression of surgically treated atypical meningiomas. *Acta Neurochir (Wien)*. 2018;160(9):1813–1822.

10. Celtikci E, Kaymaz AM, Akgul G, Karaaslan B, Emmez OH, Borcek A. Retrospective Analysis of 449 Intracranial Meningioma Patients Operated Between 2007 and 2013 at a Single Institute. *Turk Neurosurg*. 2018;28(1):1–6.

11. Unterberger A, Ng E, Pradhan A, et al. Adjuvant radiotherapy for atypical meningiomas is associated with improved progression free survival. *J Neurol Sci*. 2021;428:117590.

12. Ukai R, Wanibuchi M, Komatsu K, et al. Recurrence Interval Within 1 Year Leads to Death in Patients with Grade 2 Meningioma. *World Neurosurg*. 2020;142:e58–e65.

13. Turner CP, McLay J, Hermans IF, et al. Tumour infiltrating lymphocyte density differs by meningioma type and is associated with prognosis in atypical meningioma. *Pathology*. 2022;54(4):417–424.

14. Torres-Bayona S, Gil-Duran M, Rodriguez-Hernandez P, et al. Radiotherapy versus observation after surgical resection of atypical meningiomas. *Interdiscip Neurosurg*. 2021;25:101201.

15. Devalckeneer A, Aboukais R, Faisant M, et al. Progestin-related WHO grade II meningiomas behavior—a single-institution comparative case series. *Neurosurg Rev*. 2022;45(2):1691–1699.

16. Simonetti G, Silvani A, Tramacere I, et al. Long term follow up in 183 high grade meningioma: A single institutional experience. *Clin Neurol Neurosurg*. 2021;207:106808.

17. Sekerci Z, Oral N, Ugurluoglu O, Colpan E, Ugur A. Evaluation of Forty-Five Atypical and Malignant Meningioma Cases: Over the 12-Years Follow-Up Period. *Turk Neurosurg*. 2004;14(1-2):12–20.

18. Sadashiva N, Poyuran R, Mahadevan A, Bhat DI, Somanna S, Devi BI. Chordoid meningioma: a clinico-pathological study of an uncommon variant of meningioma. *J Neurooncol*. 2018;137(3):575–582.

19. Ros-Sanjuan A, Iglesias-Morono S, Carrasco-Brenes A, Bautista-Ojeda D, Arraez-Sanchez MA. Atypical Meningiomas: Histologic and Clinical Factors Associated With Recurrence. *World Neurosurg*. 2019;125:e248–e256.

20. Rebchuk AD, Alam A, Hounjet CD, et al. Survival and Recurrence Outcomes Following Adjuvant Radiotherapy for Grade 2 Intracranial Meningiomas: 13-Year Experience in a Tertiary-Care Center. *World Neurosurg*. 2022;161:e748–e756.

21. Press RH, Prabhu RS, Appin CL, et al. Outcomes and patterns of failure for grade 2 meningioma treated with reduced-margin intensity modulated radiation therapy. *Int J Radiat Oncol Biol Phys*. 2014;88(5):1004–1010.

22. Prat-Acin R, Guarin-Corredor MJ, Galeano-Senabre I, Ayuso-Sacido A, Vera-Sempere F. Value of KI-67/MIB-1 labeling index and simpson grading system to predict the recurrence of who grade I intracranial meningiomas compared to who grade II. *J Clin Neurosci*. 2021;86:32–37.

23. Poulen G, Vignes JR, Le Corre M, Loiseau H, Bauchet L. WHO grade II meningioma: Epidemiology, survival and contribution of postoperative radiotherapy in a multicenter cohort of 88 patients. *Neurochirurgie*. 2020;66(2):73–79.

24. Piscevic I, Villa A, Milicevic M, et al. The Influence of Adjuvant Radiotherapy in Atypical and Anaplastic Meningiomas: A Series of 88 Patients in a Single Institution. *World Neurosurg*. 2015;83(6):987–995.

25. Phonwijit L, Khawprapa C, Sitthinamsuwan B. Progression-Free Survival and Factors Associated with Postoperative Recurrence in 126 Patients with Atypical Intracranial Meningioma. *World Neurosurg*. 2017;107:698–705.

26. Pessina F, Navarria P, Clerici E, et al. Intracranial Meningiomas: A Systematic Analysis of Prognostic Factors for Recurrence in a Large Single Institution Surgical Series. *World Neurosurg*. 2019;123:e273–e279.

27. Park S, Cha YJ, Suh SH, et al. Risk group-adapted adjuvant radiotherapy for WHO grade I and II skull base meningioma. *J Cancer Res Clin Oncol*. 2019;145(5):1351–1360.

28. Park CK, Jung NY, Chang WS, Jung HH, Chang JW. Gamma Knife Radiosurgery for Postoperative Remnant Meningioma: Analysis of Recurrence Factors According to World Health Organization Grade. *World Neurosurg*. 2019;132:e399–e402.

29. Park CJ, Choi SH, Eom J, et al. An interpretable radiomics model to select patients for radiotherapy after surgery for WHO grade 2 meningiomas. *Radiat Oncol*. 2022;17(1):147.

30. Moon HS, Jung S, Jang WY, Jung TY, Moon KS, Kim IY. Intracranial Meningiomas, WHO Grade II: Prognostic Implications of Clinicopathologic Features. *J Korean Neurosurg Soc*. 2012;52(1):14–20.

31. Momin AA, Soni P, Shao J, et al. Adjuvant radiation versus observation with salvage radiation after gross-total resection of WHO grade II meningiomas: a propensity score-adjusted analysis. *J Neurosurg*. 2022;136(6):1517–1524.

32. Mattozo CA, De Salles AAF, Klement IA, et al. Stereotactic radiation treatment for recurrent nonbenign meningiomas. *J Neurosurg*. 2007;106(5):846–854.

33. Mantovani A, Di Maio S, Ferreira MJ, Sekhar LN. Management of Meningiomas Invading the Major Dural Venous Sinuses: Operative Technique, Results, and Potential Benefit for Higher Grade Tumors. *World Neurosurg*. 2014;82(3-4):455–467.

34. Liang Y, Ning B, Hua X, et al. Atypical meningioma: a retrospective analysis of six cases and literature review. *Transl Cancer Res*. 2021;10(3):1509–1518.

35. Lee G, Lamba N, Niemierko A, et al. Adjuvant Radiation Therapy Versus Surveillance After Surgical Resection of Atypical Meningiomas. *Int J Radiat Oncol Biol Phys*. 2021;109(1):252–266.

36. Di Ieva A, Laiq S, Nejad R, et al. Chordoid meningiomas: incidence and clinicopathological features of a case series over 18 years. *Neuropathology*. 2015;35(2):137–147.

37. Kumar N, Kumar R, Khosla D, Salunke PS, Gupta SK, Radotra BD. Survival and failure patterns in atypical and anaplastic meningiomas: A single-center experience of surgery and postoperative radiotherapy. *J Cancer Res Ther*. 2015;11(4):735–739.

38. Korshunov A, Shishkina L, Golanov A. DNA topoisomerase II-alpha and cyclin A immunoexpression in meningiomas and its prognostic significance: an analysis of 263 cases. *Arch Pathol Lab Med*. 2002;126(9):1079–1086.

39. Ko KW, Nam DH, Kong DS, Lee JI, Park K, Kim JH. Relationship between malignant subtypes of meningioma and clinical outcome. *J Clin Neurosci*. 2007;14(8):747–753.

40. Klinger DR, Flores BC, Lewis JJ, et al. Atypical Meningiomas: Recurrence, Reoperation, and Radiotherapy. *World Neurosurg*. 2015;84(3):839–845.

41. Kim MS, Kim KH, Lee EH, et al. Results of immunohistochemical staining for cell cycle regulators predict the recurrence of atypical meningiomas. *J Neurosurg*. 2014;121(5):1189–1200.

42. Morokoff AP, Zauberman J, Black PM. Surgery for convexity meningiomas. *Neurosurgery*. 2008;63(3):427–434.

43. Nowak A, Dziedzic T, Krych P, Czernicki T, Kunert P, Marchel A. Benign versus atypical meningiomas: risk factors predicting recurrence. *Neurol Neurochir Pol*. 2015;49(1):1–10.

44. Karabagli P, Karabagli H, Mavi Z, Demir F, Ozkeles EY. Histopathological and Clinical Features as Prognostic Factors of Atypical Meningiomas. *Turk Neurosurg*. 2020;30(5):746–575.

45. Kalasauskas D, Kronfeld A, Renovanz M, et al. Identification of high-risk atypical meningiomas according to semantic and radiomic features. *Cancers (Basel)*. 2020;12(10):1–11.

46. Joseph E, Sandhyamani S, Rao MB, Nair S, Radhakrishnan VV. Atypical meningioma: a clinicopathological analysis. *Neurol India*. 2000;48(4):338–342.

47. James Z, Makwana M, Hayhurst C. De Novo Skull Base Atypical Meningioma: Incidence and Outcome. *J Neurol Surg B Skull Base*. 2023;84(2):113–118.

48. Holleczek B, Zampella D, Urbschat S, et al. Incidence, mortality and outcome of meningiomas: A population-based study from Germany. *Cancer Epidemiol*. 2019;62:101562.

49. Hammouche S, Clark S, Wong AHL, Eldridge P, Farah JO. Long-term survival analysis of atypical meningiomas: survival rates, prognostic factors, operative and radiotherapy treatment. *Acta Neurochir (Wien)*. 2014;156(8):1475–1481.

50. Halliday J, Fernandes H. Meningioma recurrence: the efficacy and cost-effectiveness of current screening. *Br J Neurosurg*. 2010;24(1):55–61.

51. Gurses ME, Zengin HY, Shikhaliyeva A, Askun CS, Mut M. Early Postoperative Adjuvant Radiotherapy Versus Active Monitoring After Gross Total Resection for Atypical Meningiomas: Factors Associated with Early Recurrence. *Turk Neurosurg*. 2023;33(4):635–641.

52. Goyal LK, Suh JH, Mohan DS, Prayson RA, Lee J, Barnett GH. Local control and overall survival in atypical meningioma: a retrospective study. *Int J Radiat Oncol Biol Phys*. 2000;46(1):57–61.

53. Gousias K, Schramm J, Simon M. The Simpson grading revisited: aggressive surgery and its place in modern meningioma management. *J Neurosurg*. 2016;125(3):551–560.

54. Fernandez C, Nicholas MK, Engelhard HH, Slavin KV, Koshy M. An analysis of prognostic factors associated with recurrence in the treatment of atypical meningiomas. *Adv Radiat Oncol*. 2016;1(2):89–93.

55. Escribano Mesa JA, Alonso Morillejo E, Parron Carreno T, et al. Risk of Recurrence in Operated Parasagittal Meningiomas: A Logistic Binary Regression Model. *World Neurosurg*. 2018;110:e112–e118.

56. Endo T, Narisawa A, Ali HSM, et al. A study of prognostic factors in 45 cases of atypical meningioma. *Acta Neurochir (Wien)*. 2016;158(9):1661–1667.

57. Di Maio S, Ramanathan D, Garcia-Lopez R, et al. Evolution and future of skull base surgery: The paradigm of skull base meningiomas. *World Neurosurg*. 2012;78(3):260–275.

58. Choi Y, Lim DH, Yu JI, et al. Prognostic Value of Ki-67 Labeling Index and Postoperative Radiotherapy in WHO Grade II Meningioma. *Am J Clin Oncol*. 2018;41(1):18–23.

59. Barresi V, Cerasoli S, Vitarelli E, Tuccari G. Density of microvessels positive for CD105 (endoglin) is related to prognosis in meningiomas. *Acta Neuropathol*. 2007;114(2):147–156.

60. Andric M, Dixit S, Dubey A, Jessup P, Hunn A. Atypical meningiomas - A case series. *Clin Neurol Neurosurg*. 2012;114(6):699–702.

61. Aghi MK, Carter BS, Cosgrove GR, et al. Long-term recurrence rates of atypical meningiomas after gross total resection with or without postoperative adjuvant radiation. *Neurosurgery*. 2009;64(1):56–60.

62. Aboukais R, Zairi F, Le Rhun E, Lejeune JP, Devos P, Reyns N. Radiation-associated grade 2 meningiomas: A nine patient-series and review of the literature. *Clin Neurol Neurosurg*. 2015;136:10–14.

63. Lee SH, Lee EH, Sung KS, Kim DC, Kim YZ, Song YJ. Ki67 Index Is the Most Powerful Factor for Predicting the Recurrence in Atypical Meningioma: Retrospective Analysis of 99 Patients in Two Institutes. *J Korean Neurosurg Soc*. 2022;65(4):558–571.

64. Lee KD, DePowell JJ, Air EL, Dwivedi AK, Kendler A, McPherson CM. Atypical meningiomas: is postoperative radiotherapy indicated? *Neurosurg Focus*. 2013;35(6):E15.

65. Unteroberdorster M, Michel A, Darkwah Oppong M, et al. The 2016 Edition of the WHO Classification of Primary Brain Tumors: Applicable to Assess Individual Risk of Recurrence in Atypical Meningioma? A Single-Center Experience. *J Neurol Surg A Cent Eur Neurosurg*. 2021;82(5):417–423.

66. Chang WI, Byun HK, Lee JH, et al. Novel Postoperative Serum Biomarkers in Atypical Meningiomas: A Multicenter Study. *Neurosurgery*. 2023;93(3):599–610.

67. Torp SH, Lindboe CF, Gronberg BH, Lydersen S, Sundstrom S. Prognostic significance of Ki-67/MIB-1 proliferation index in meningiomas. *Clin Neuropathol*. 2005;24(4):170–174.

68. Sun SQ, Kim AH, Cai C, et al. Management of Atypical Cranial Meningiomas, Part 1: Predictors of Recurrence and the Role of Adjuvant Radiation After Gross Total Resection. *Neurosurgery*. 2014;75(4):347–355.

69. Shan B, Zhang J, Song Y, Xu J. Prognostic factors for patients with World Health Organization grade III meningiomas treated at a single center. *Medicine (Baltimore)*. 2017;96(26):e7385.

70. Ramina R, Neto MC, Fernandes YB, Aguiar PHP, De Meneses MS, Torres LFB. Meningiomas of the jugular foramen. *Neurosurg Rev*. 2006;29(1):55–60.

71. Kim EY, Weon YC, Kim ST, et al. Rhabdoid Meningioma: Clinical Features and MR Imaging Findings in 15 Patients. *AJNR Am J Neuroradiol*. 2007;28(8):1462–1465.

72. Kim EY, Kim ST, Kim HJ, Jeon P, Kim KH, Byun HS. Intraventricular meningiomas: radiological findings and clinical features in 12 patients. *Clin Imaging*. 2009;33(3):175–180.

73. Ildan F, Erman T, Gocer AI, et al. Predicting the probability of meningioma recurrence in the preoperative and early postoperative period: A multivariate analysis in the midterm follow-up. *Skull Base*. 2007;17(3):157–171.

74. Li B, Tao B, Bai H, et al. Papillary meningioma: an aggressive variant meningioma with clinical features and treatment: a retrospective study of 10 cases. *Int J Neurosci*. 2016;126(10):878–887.
